# Genome-wide fitness analyses of the foodborne pathogen *Campylobacter jejuni* in *in vitro* and *in vivo* models

**DOI:** 10.1038/s41598-017-01133-4

**Published:** 2017-04-28

**Authors:** Stefan P. de Vries, Srishti Gupta, Abiyad Baig, Elli Wright, Amy Wedley, Annette Nygaard Jensen, Lizeth LaCharme Lora, Suzanne Humphrey, Henrik Skovgård, Kareen Macleod, Elsa Pont, Dominika P. Wolanska, Joanna L’Heureux, Fredrick M. Mobegi, David G. E. Smith, Paul Everest, Aldert Zomer, Nicola Williams, Paul Wigley, Thomas Humphrey, Duncan J. Maskell, Andrew J. Grant

**Affiliations:** 1 Department of Veterinary Medicine, University of Cambridge, Cambridge, United Kingdom; 2 Department of Infection Biology, Institute of Infection and Global Health, University of Liverpool, Leahurst Campus, Neston United Kingdom; 3 Technical University of Denmark, National Food Institute, Copenhagen, Denmark; 4 Department of Agroecology, University of Aarhus, Slagelse, Denmark; 5 University of Glasgow, Veterinary School, Glasgow, United Kingdom; 6 Department of Paediatric Infectious Diseases, Radboud Institute for Molecular Life Sciences, Radboud University Medical Centre, Nijmegen, The Netherlands; 7 Heriot-Watt University, School of Life Sciences, Edinburgh, Scotland United Kingdom; 8 Department of Infectious Diseases and Immunology, Faculty of Veterinary Medicine, Utrecht University, Utrecht, The Netherlands; 9 Department of Epidemiology and Population Health, Institute of Infection and Global Health, University of Liverpool, Leahurst Campus, Neston United Kingdom; 10 School of Medicine, Institute of Life Sciences, Swansea University, Swansea, United Kingdom; 11 School of Veterinary Medicine and Science, University of Nottingham, Sutton Bonnington, Leicestershire United Kingdom; 12 Institute of Infection, Immunity and Inflammation, University of Glasgow, Glasgow, United Kingdom; 13 Division of Molecular Carcinogenesis, The Netherlands Cancer Institute, Amsterdam, The Netherlands

## Abstract

*Campylobacter* is the most common cause of foodborne bacterial illness worldwide. Faecal contamination of meat, especially chicken, during processing represents a key route of transmission to humans. There is a lack of insight into the mechanisms driving *C*. *jejuni* growth and survival within hosts and the environment. Here, we report a detailed analysis of *C*. *jejuni* fitness across models reflecting stages in its life cycle. Transposon (Tn) gene-inactivation libraries were generated in three *C*. *jejuni* strains and the impact on fitness during chicken colonisation, survival in houseflies and under nutrient-rich and –poor conditions at 4 °C and infection of human gut epithelial cells was assessed by Tn-insertion site sequencing (Tn-seq). A total of 331 homologous gene clusters were essential for fitness during *in vitro* growth in three *C*. *jejuni* strains, revealing that a large part of its genome is dedicated to growth. We report novel *C*. *jejuni* factors essential throughout its life cycle. Importantly, we identified genes that fulfil important roles across multiple conditions. Our comprehensive screens showed which flagella elements are essential for growth and which are vital to the interaction with host organisms. Future efforts should focus on how to exploit this knowledge to effectively control infections caused by *C*. *jejuni*.

## Introduction

Infection by *Campylobacter* is the most common cause of foodborne bacterial diarrhoeal disease worldwide, responsible for ~96 million foodborne illnesses and ~21,000 foodborne deaths in 2010^1^. While most cases are self-limiting, for some, campylobacteriosis is a particularly serious infection, and it is also associated with severe post-infection complications, including irritable bowel and Guillian-Barré syndromes. Consumption of undercooked poultry, unpasteurised dairy products and contaminated water represent the most common sources of human infection^2, 3^. *Campylobacter jejuni* has a broad range of environmental reservoirs that include water, birds and other domestic animals^3^. In addition, flies have been implicated as a transmission vector for *C*. *jejuni* for both chicken flocks and possibly for humans^4–7^.

*C*. *jejuni* encounters and has to overcome various stress conditions whilst passing through the gastrointestinal tract of humans and other animals, during processing of food products (*e*.*g*. slaughter process of poultry), on/in food (*e*.*g*. in milk or poultry meat, generally stored at low temperature) and in the environment (*e*.*g*. in surface water, soil, or in flies, the latter representing a transmission vector)^4–8^. However, compared to other enteric pathogens such as pathogenic *Escherichia coli* and *Salmonella* spp, the survival mechanisms used by *C*. *jejuni* to cope with these stresses are less well-understood^9^.

Based on genome analysis, the capacity of *C*. *jejuni* to survive outside the host and adapt to environmental stress conditions appears to be limited due to the lack of key stress regulators found in other enteric pathogens^10^. Colonisation and infection of host organisms by *C*. *jejuni* is a multifactorial process with key roles for “swimming” motility, chemotaxis, interaction with gut epithelial cells, toxin production, and oxidative and metabolic stress adaptation^11, 12^.

In this study, we screened extensive *C*. *jejuni* transposon (Tn) gene inactivation mutant libraries to comprehensively assess which genes are required during *in vitro* growth, chicken colonisation and during exposure to low temperature in nutrient-rich and –poor conditions, and infection of human gut epithelial cells. This study reinforces the importance of flagella for host interactions, and identifies genes required for survival, colonisation and infection in multiple phases of the bacterium’s life cycle.

## Results

### Identification of genes required for fitness

**Figure 1:**
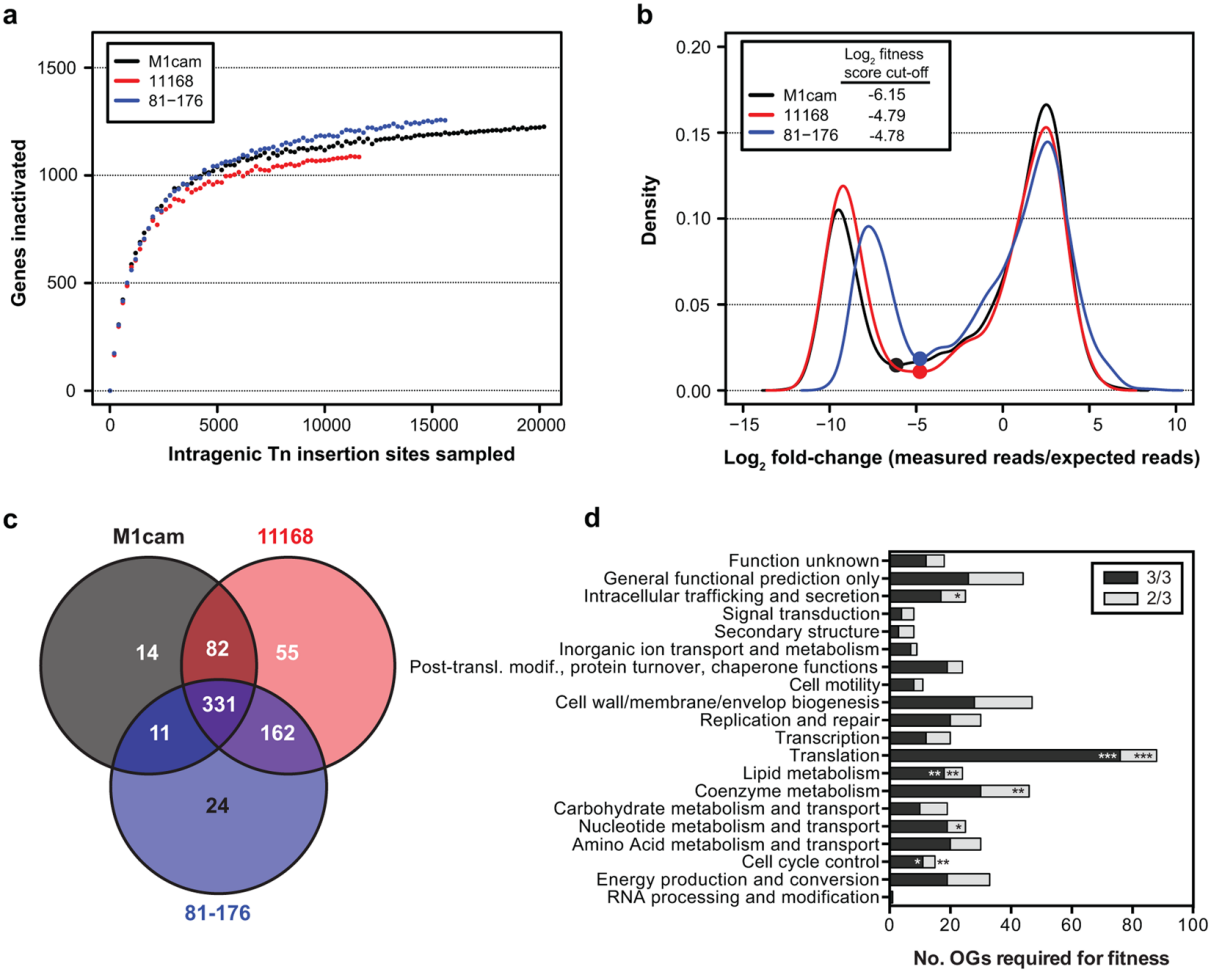
Gene fitness analysis during *in vitro* growth of *C*. *jejuni* M1cam, 11168 and 81–176. (**a**) Rarefaction analysis of intragenic Tn insertions. (**b**) Density plot fitness score (Log_2_ fold-change measured reads/expected reads) per gene. Dots indicate fitness score cut-off values. (**c**) Overlap of homologous gene (HG) clusters (HGs) required for fitness in *C*. *jejuni* M1cam, 11168, and 81–176, representing the “pan-fitness genome”. HG clusters that are required for fitness in one or more strains are displayed. (**d**) Functional class (COG; Cluster of Orthologous Genes) enrichment analysis of fitness genes. Fisher exact test with *Q*-value multiple testing correction; *Q < 0.05, **Q < 0.01 and ***Q < 0.001.

To assess the genetic basis of *C*. *jejuni* growth and survival, genes were randomly inactivated using Tn mutagenesis in three well-characterized *C*. *jejuni* strains [M1cam^13, 14^, NCTC 11168 (hereafter referred to as 11168)^10^ and 81–176^15^]. Tn mutant libraries were characterised by Tn insertion site sequencing (Tn-seq^16^) (Table S1), providing a measure for the relative abundance of each Tn mutant in the library. Genes that are required for growth and survival, hereafter referred to as “fitness” genes, cannot tolerate Tn insertions, or Tn mutants in these genes are severely underrepresented in the libraries. To identify fitness genes, 23,334 unique chromosomal Tn insertions were analysed in M1cam, 15,008 in 11168, and 17,827 in 81–176 (Table S1), reaching near-saturation in terms of the number of genes that could be inactivated (Fig. 1a). In addition to chromosomal Tn insertions, 2,009 and 1,919 unique insertions were in the 81–176 plasmids pVir^17^ and pTet^18^, respectively (Table S1). No apparent Tn insertion bias was observed (Fig. S1) and each Tn library predominately yielded unique Tn insertions (Fig. S2).

Gene fitness score (Log_2_ fold-change between the observed *vs* expected sequence reads^19^) density plots followed a bimodal distribution with the “left” population representing genes required for *in vitro* growth and survival (Fig. 1b). In total, 445 genes were required for fitness in M1cam, 413 genes in 81–176 and 499 in 11168 (Table S2). Interestingly, *cpp13* in the pTet plasmid in strain 81–176 appeared to contribute to fitness. A variant of *C*. *jejuni* 81–176 that has lost pTet exists (D. Hendrixson, personal communication), which implies that *cpp13* is not obligate essential but may contribute to fitness, or it could be antitoxin of an uncharacterised toxin-antitoxin system. Unexpectedly, Tn insertions were observed in *dnaA* (1,323 bp) at bp position 352 in M1cam and at position 1,115 in 81–176. Consequently, *dnaA* did not pass the stringent fitness gene criteria. Disruption of *dnaA* may be tolerated at the 3′ end of the gene, in the rare event of a secondary site mutation^20^, or due to the existence of merodiploids^21^.

To comprehensively assess fitness genes in the species *C*. *jejuni*, homologous genes (HG) were compared for the three *C*. *jejuni* strains (Table S2). Out of the 1,424 identified HG clusters, M1cam has members in all HG clusters, and 81–176 and 11168 have gene members in 1,423 HG clusters, indicating that in terms of functional capacity the three tested *C*. *jejuni* strains are highly related. 331 HG clusters were required for fitness in all three strains tested and 486 HG clusters were required in two or more strains (Fig. 1c), indicating that a large part of the *C*. *jejuni* genome is dedicated to growth and survival under the condition tested. We found that 845 HG clusters were not required for fitness in any of the analysed strains. Genes implicated in fitness were relatively dispersed across the *C*. *jejuni* genomes, but there were regions that were (almost) devoid of fitness genes, for example, the flagellar glycosylation gene cluster (Fig. S3). The number of fitness HG clusters shared by *C*. *jejuni* M1cam, 81–176 and 11168 was substantially larger, at 331, than the 175–233 genes previously reported to be obligatory essential^22–25^. This may be partially due to the inclusion of genes, in our study, whose inactivation is lethal (obligate essential) as well as genes which when inactivated by a Tn result in severely compromised growth and/or survival. Further, this could be related to *C*. *jejuni* strain differences, growth conditions, the Tn element used, the number of Tn mutants analysed and the read-out technology. A systematic review of the literature on *C*. *jejuni* 11168 defined gene deletion and Tn mutants revealed that for 38 out of 486 (7.8%) fitness HG clusters (required in two or more strains) identified in this study, mutants have been reported, indicating a low false-positive rate in our study. Although mutants in some of these genes have a growth defect, *e*.*g*. as reported for a *C*. *jejuni* 11168 *pycB* mutant^26^, especially when compared against other ‘more-fit’ mutants or mutants with wild-type fitness, as is the case in the Tn screen.

Genes implicated in translation, lipid metabolism and cell cycle control were overrepresented amongst the genes required for fitness during *in vitro* growth/survival in all three tested *C*. *jejuni* strains. Extending the analysis to fitness genes required in two or more strains also showed overrepresentation of coenzyme metabolism, nucleotide metabolism and intracellular trafficking and secretion genes (Fig. 1d). Required for fitness were, amongst others, genes implicated in replication, transcription, translation (40 out of 49 ribosomal protein genes), purine and pyrimidine metabolism, energy metabolism (ATP and NAD synthase, NADH-quinone oxidoreductase), isoprene biosynthesis, protein secretion (Sec and Tat pathway), as well as genes involved in cofactor biosynthesis (thiamine, folic acid and heme) and oxidative stress (see Table S2 for a complete overview). Further, the complete gluconeogenic pathway was found to be required for fitness whereas the majority of the enzymes of the tricarboxylic acid (TCA) cycle were not, except for aconitase (*acnB*), probably reflecting flexibility in this part of the bacterium’s metabolism.

*C*. *jejuni* expresses surface structures such as flagella, lipooligosaccharide (LOS) and capsular polysaccharides (CPS). Inactivation of genes in these pathways severely attenuated fitness. This included flagellar basal body rod proteins (encoded by *flgAC* and *fliEL*), the MS-ring (*fliF*) and C-ring (*fliG*) as well as components of the flagellar type III secretion system (*fliQH*). Genes required for formation of the LOS lipid A molecule (*lpxABCDL*), KDO (*kdsAB*) and the first L-*glycero*-D-*manno*-heptose residue (*waaC*) were required for fitness, whereas the remainder of the genes responsible for the core oligosaccharide were not. Of the capsular biosynthesis gene cluster only the inactivation of the last two genes (*kpsDF*) resulted in impaired fitness. Genes required for cell envelope generation were also important for fitness including fatty acid biosynthesis genes (*accABCD* and *fabDFGHLZ*), peptidoglycan (*dapADEF*, *ddl*, *murABCDEFG*, *pbpABC*), and the rod-shape determining protein genes (*mreBCD*). Protein glycosylation is tightly linked to virulence, of which the *N*-linked protein glycosylation pathway genes *pglACD* were required for fitness. This is in contrast to flagella glycosylation genes, which had no conserved role in fitness during *in vitro* growth and survival (Table S1).

### Quantitative analysis of genes implicated in the life cycle of *C*. *jejuni*

**Figure 2:**
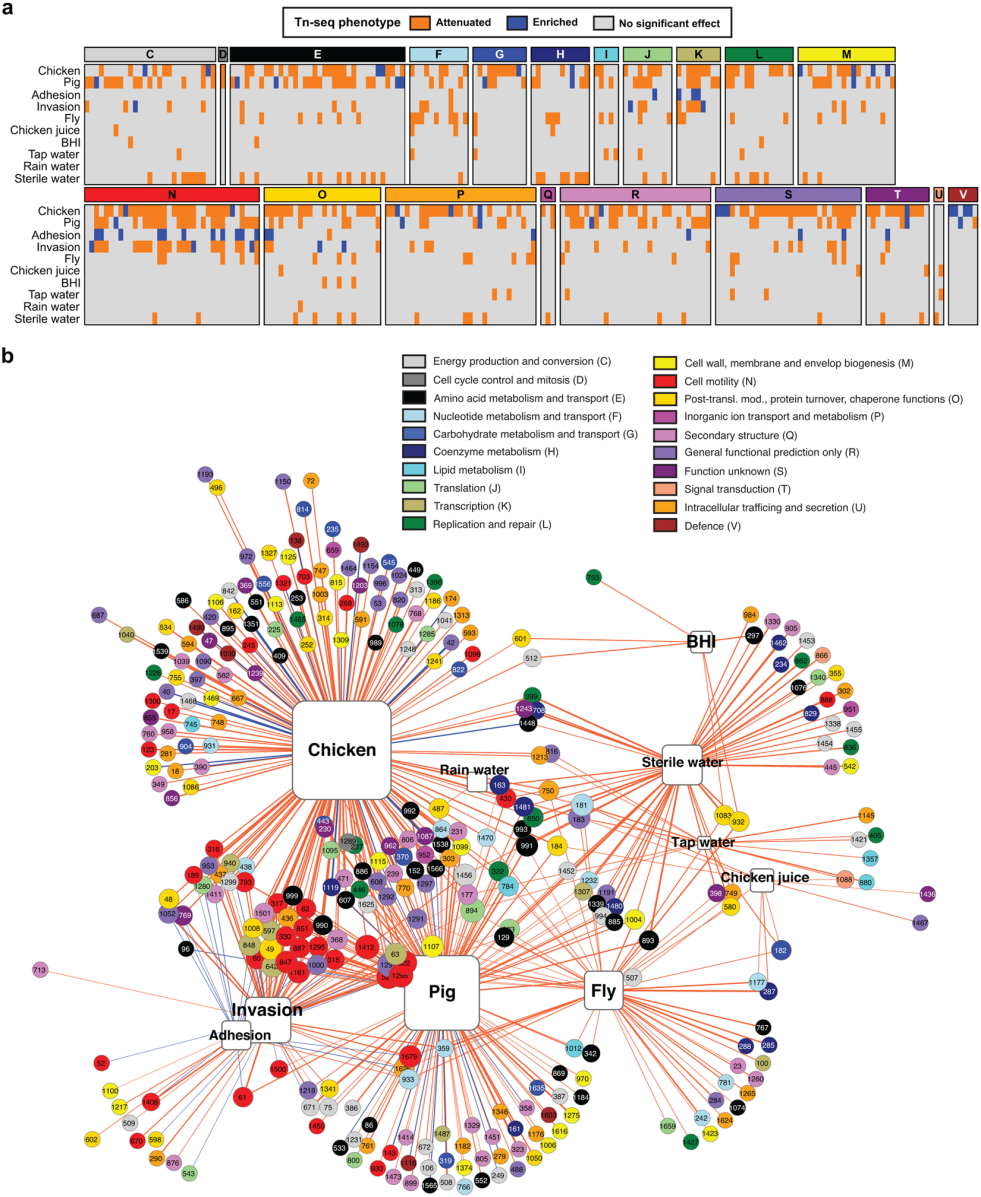
Identification of conditional essential genes in *C*. *jejuni*. (**a**) Effect of Tn insertions on the ability of *C*. *jejuni* M1cam to colonize commercial broiler chickens, infect gnotobiotic piglets, adhere and invade gut epithelial tissue culture cells, survive in flies and at 4 °C in various media (chicken juice, BHI, tap water, rain water, and sterile water). Genes of which Tn mutants showed significantly attenuated or enriched fitness in the experimental models are shown and are grouped according to their COG functional classification. Data represented as Log_2_ fold-change is also presented in Table S4. (**b**) Gene-model interaction network showing attenuated (orange lines) and enriched (blue lines) Tn-seq scores linked to their respective models; the thickness of the connecting lines corresponds to the Log_2_ fold-change (input/output). The gene numbers correspond to the *C*. *jejuni* M1cam locus-tags^13^ and are color-coded according to their COG functional class. The size of the genes and the models displayed increases with the number of interactions.

The same extensive Tn library in strain M1cam (Table S1; 9,951 unique Tn insertions with 1,124 genes harbouring Tn insertions) was screened in various *in vivo* and *in vitro* models as a proxy for some of the conditions that *C*. *jejuni* might encounter during its life cycle, facilitating a comparative analysis across the models. The M1cam library was screened during the colonisation of commercial broiler chickens (natural host), survival in the housefly (transmission vector), survival under nutrient-rich- and nutrient-poor conditions at low temperature, and in models that mimic (stages of) infection of humans, *i*.*e*. adhesion and invasion of human gut epithelial cells. For comparative purposes we included data that we obtained in a study analysing the infection of gnotobiotic piglets (de Vries *et al*., submitted). Tn-seq analysis after exposure to a challenge, compared with the control condition, provided a quantitative measure for the contribution of a gene to fitness in each of the models (Fig. 2 and Tables S3 and S4). The number of Tn mutants recovered from each model confirmed that the complexity of the Tn library was maintained in all models, however library complexity was reduced after colonisation of chickens, *i*.*e*. 23% of the input Tns were recovered from chickens (Fig. S4). Therefore, we applied more stringent criteria for selection of candidate genes required in this model (Table S3 and Methods). A detailed analysis for each of the models in this study is provided below.

### Genes required for chicken colonisation

Broilers generally become colonised with *Campylobacter* spp. at about 3–4 weeks of age^27^. We have screened the M1cam Tn library ‘C’ in a relevant chicken colonisation model, *i*.*e*. in 3-week-old Ross 308 commercial broiler birds. Four cages, each with 5–7 birds, were inoculated with the library and 6 days post-inoculation (p.i.) colonising Tn mutants were recovered. Due to the coprophagic behaviour of chickens, each individual cage was considered a colonisation unit^28^. We hypothesised that a group-level approach would improve the robustness of the analysis and reduce any bias introduced by random dropout of Tn mutants as observed in our previous work using a signature-tagged mutagenesis approach^29^ and using wild-type isogenic-tagged strains (WITS) that have indistinguishable phenotypes in pure culture^30^. On average, cages harboured 1,641 ± 226 Tn mutants (>10 reads) compared to 7,325 ± 538 Tns in the input (Fig. S4), revealing a population bottleneck, even at the cage level. However, the average read count of the four cages demonstrated recovery of 3,111 Tn mutants (>10 reads) covering 701 genes. This indicated that, with additional stringent filtering steps (see Methods) a large part of the genome could be analysed.

Tn mutants of 172 genes were significantly under-represented in colonised chickens, whereas 24 appeared to enhance fitness during colonisation (Fig. 2 and Tables S3 and S4). Genes linked to the COG class “cell motility” were significantly enriched amongst those required during colonisation (Fig. 2 and Fig. S5), underscoring the prominent role of motility for colonisation of chickens. Previously, Johnson *et al*., screened 1,155 *C*. *jejuni* 81–176 Tn mutants in 1-day-old chicks and identified 130 genes that were required for colonisation and 30 genes that appeared to enhance colonisation^23^. However, interpretation of the importance of these colonisation genes is complicated due to limited validation; only the importance of *mapA* was confirmed^23^. We found a limited overlap of 23 chicken colonisation genes between the two studies, 11 of which were linked to motility and the flagellar system. The datasets had one gene (CJM1_0420; hypothetical protein) in common that was beneficial for colonisation. Differences between identified colonisation genes are likely the result of the model employed, *e*.*g*. older birds harbouring a more mature intestinal microbial community and younger birds being more permissive for colonisation by *C*. *jejuni*, *e*.*g*. as reflected in a lower inoculation dose to establish colonisation^31^.

**Figure 3:**
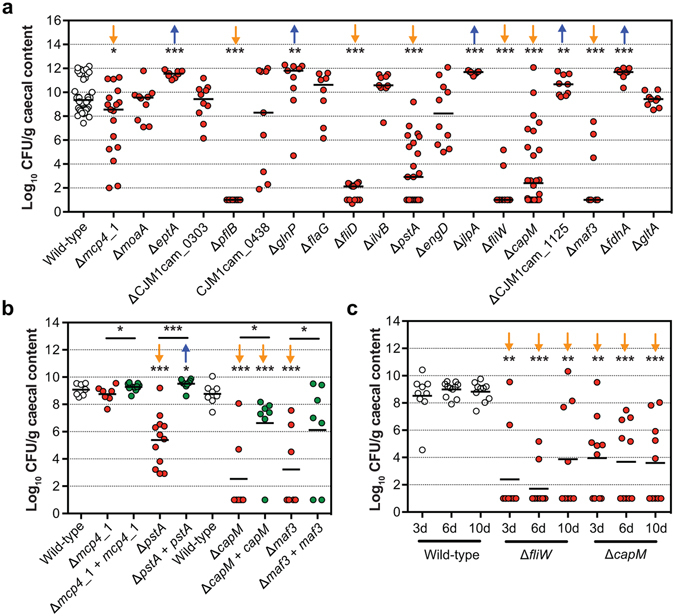
Validation of chicken colonisation Tn mutant library screen. (**a**) Colonisation levels of *C*. *jejuni* M1cam gene deletion mutants 6 d pi. (*n* ≥ 7) (**b**) Colonisation of gene deletion mutants and complemented gene deletion mutants (*n* ≥ 7). (**c**) Colonisation kinetics of *C*. *jejuni* M1cam wild-type and defined mutants in *fliW* and *capM* (*n* ≥ 9). Chickens being colonised *vs* not colonised were 2/8, 2/8 and 3/6 for the *fliW* mutant and 4/5, 4/6 and 5/5 for the *capM* mutant at day 3, 6 and 10 p.i., respectively. Statistical significance was analysed using a Mann-Whitney test with *P < 0.05, **P < 0.01 and ***P < 0.001. The orange arrows represent an attenuated phenotype while the blue arrows represent an enriched chicken colonisation phenotype.

For validation, 19 genes were tested individually for their ability to colonise chickens. The importance in chicken colonisation was confirmed for genes involved in chemotaxis (*mcp4_1*), the flagellar system (*pflB*, *fliD*, *fliW*, and *maf3*), *N*-linked protein glycosylation system (*capM*/*pglH*), and phosphate transport (*pstA*) (Fig. 3a). Although motility is considered a driving factor in chicken colonisation, the colonisation deficient *maf3*, *capM* and *pstA* displayed wild-type motility (Fig. S6). In the screen, mutants in *eptA* (also referred to as *eptC* ^32^), *glnP*, *jlpA*, *fdhA* and CJM1cam_1125 showed reduced colonisation. However, deletion mutants in these genes colonised at slightly higher levels compared to the wild-type (Fig. 3a); motility of these gene deletion mutants did not differ significantly from the wild-type (Fig. S6). No difference to the wild-type was found in the colonisation proficiency of deletion mutants in *moaA*, CJM1cam_0303 (hypothetical protein), CJM1cam_0438 (hypothetical protein), *flaG*, *ilvB*, *engD* and *gltA*. Identification of these genes in the Tn library screen might be attributed to the competition effect of other Tn mutants in the screen, whilst validation experiments were conducted with single mutant inocula. We opted for validation experiments with single mutant inocula due to the unpredictable outcome of competition experiments, even in the simplest case of two phenotypically indistinguishable WITS^30^. It is possible that some candidate colonisation genes represent false positives due to the random loss of mutants, *i*.*e*. an infection bottleneck that was observed in the chicken model.

Genetic complementation of *pstA*, *capM* and *maf3* did significantly increase their colonisation capacity compared to their respective gene deletion mutants (Fig. 3b), confirming their role in chicken colonisation. Attempts to complement *fliD* (encoding the flagellar cap protein) failed (Fig. S8). When analysing colonisation of the genetically complemented *mcp4_1* mutant in chickens, we found that, in contrast to initial validation experiments (Fig. 3a), the *mcp4_1* mutant colonised chickens; which could be the result of intrinsically lower colonisation resistance in the batch of chickens used in this experiment. Despite the existence of a colonisation bottleneck and the incomplete reproducibility when comparing defined deletion mutants with the results obtained in the Tn library screen, this work has identified *pstA*, *capM*, *maf3* and *mcp4*_1 as novel colonisation factors in 3-week-old broiler chickens.

In validation experiments (Fig. 3a) we observed an ‘all or nothing’ colonisation effect in some chickens infected with particular defined mutants, *e*.*g*. *pflA*, *fliD* and *fliW*, whereas for other deletion mutants, such as *capM* and *mcp4_1*, colonisation levels varied considerably between the birds. This indicates that there might be differences in the colonisation permissiveness/resistance between birds, within and between experiments. To investigate this in more detail, the colonisation of the wild-type, *fliW* and *capM* mutants were measured at different time intervals (3, 6 and 10 days p.i.) (Fig. 3c). Chickens were colonised at high levels from 3 days onwards and neither an increase in the levels of colonisation nor the number of colonised birds was observed. Surprisingly, the level of colonisation of the *fliW* and *capM* mutants did not increase between 3 and 10 days p.i. and there was also no obvious increase in the number of colonised chickens (Fig. 3c). We hypothesise that the colonisation responses observed in our validation experiments were potentially confounded by variation in gut microbiota composition or differential inflammatory responses elicited during colonisation^33–35^.

### Genes required for survival in the housefly

**Figure 4:**
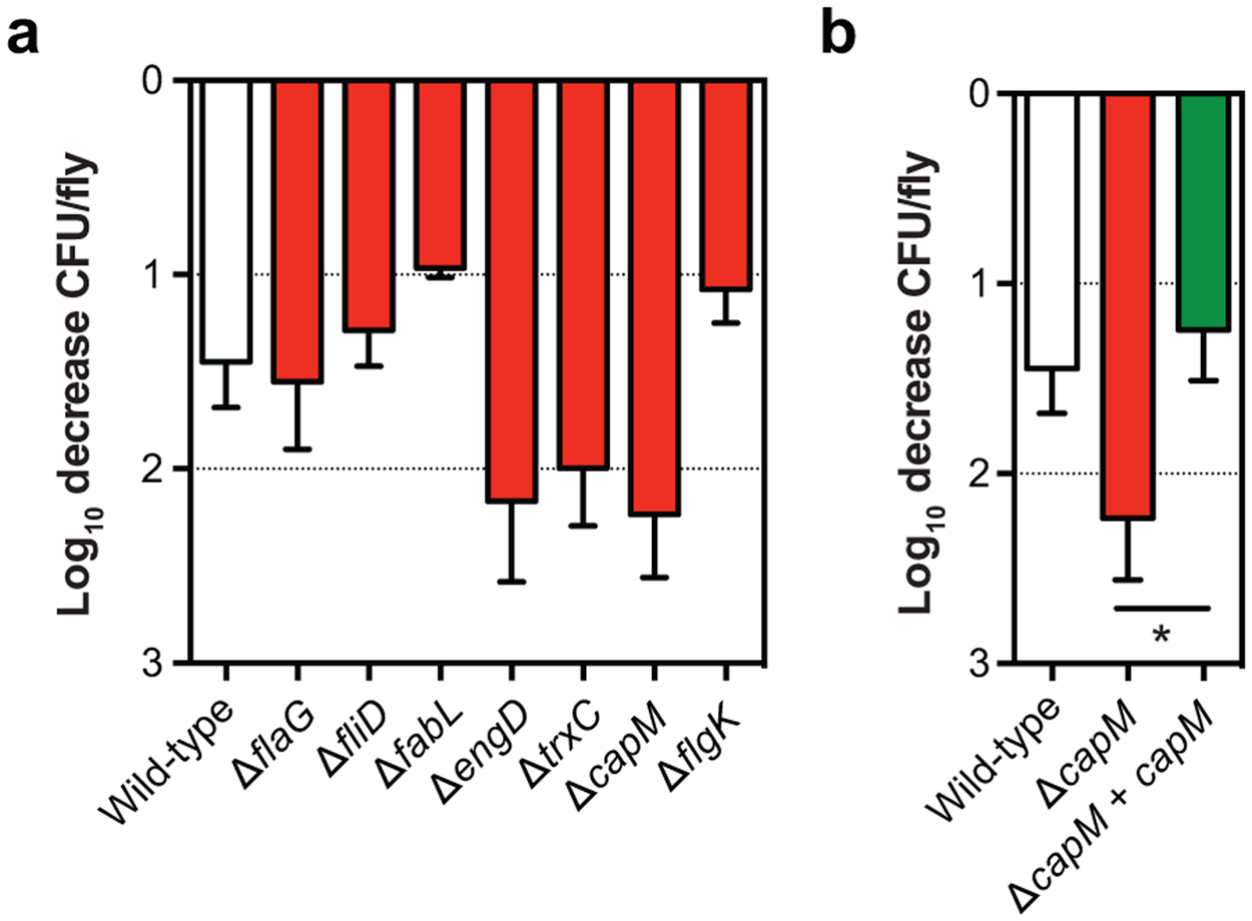
Validation of Tn mutant library screen during survival in the housefly. (**a**) Survival (Log_10_ decrease inoculum *vs* recovered) of defined *C*. *jejuni* M1 gene deletion mutant in the housefly after 4 h. (**b**) Survival of wild-type, *capM* defined gene deletion mutant and genetically complemented mutant. Data shown are Log_10_ decrease of CFU per fly relative to the inoculum and plotted as means with SEM (*n* ≥ 4). Significance was analysed using a Mann-Whitney test with *P < 0.05.

As a transmission vector model for *C*. *jejuni* ^4–7^, survival of the M1cam Tn library ‘C’ was examined 4 h after individual inoculation of houseflies. Tn mutants in 48 genes showed reduced survival and no genes were identified for enhanced survival (Fig. 2 and Tables S3 and S4). Genes of the COG class “nucleotide transport and metabolism” were overrepresented amongst the genes linked to survival in the housefly (Fig. 2 and Fig. S5). These were the non-essential genes in the purine (*purLMN*) and pyrimide (*pyrC*_1/2 and *pyrDF*) biosynthesis pathways. Although the Tn library screen identified a number of candidate survival genes, validation experiments with 7 defined gene deletion mutants (inoculated as single mutants) were non-confirmative (Fig. 4a). Our inability to confirm the role of identified candidate genes might be the result of low levels of attenuation, or due to the lack of competition with other mutants. However, the attenuated survival of a *capM* deletion mutant approached significance (*P* = 0.0513, two-tailed Mann-Whitney). As the importance of *capM* in chicken colonisation was confirmed *via* genetic complementation, we also tested the *capM* mutant alongside its genetically complemented mutant for survival in the housefly, which confirmed that *capM* is also involved in survival in the housefly (Fig. 4b).

### Survival in nutrient-rich and –poor conditions at low temperature

The M1cam Tn library ‘C’ was incubated at 4 °C in “chicken juice” (liquid obtained after thawing chicken carcasses), BHI broth and in water (sterile, tap and rain), with the “water” models being included to assess the survival under more general environmental conditions. Tn mutants that survived after 7 days incubation were compared to the Tn library composition at T = 0 days. Ten genes were implicated in survival in chicken juice and 6 genes in BHI medium. Considerable variation was found for the number of genes implicated in survival at 4 °C in nutrient-poor conditions, with 13 genes identified in tap water and 57 in sterile water. However, only one candidate gene, encoding the heat shock response protein ClpB, was identified as being required for survival in rain water (Fig. 2 and Tables S3 and S4). These variations could arise due to differences in chemical composition, pH and potentially other bacteria present in water samples^36, 37^. Given the relatively low number of genes associated with survival at low temperature and the relatively mild attenuation, as expressed by Tn-seq fitness score, we hypothesise that survival may be a passive rather than active mechanism^38^.

At low temperature, an oxidative stress response is induced in *C*. *jejuni* ^9, 39^. In our Tn library screen, the gene encoding the oxidoreductase TrxC was found to be required for survival in sterile water and BHI at low temperature. In addition, the regulator of oxidative stress, PerR, was linked to survival in sterile water. PerR plays a role in controlling oxidative stress resistance and survival under aerobic conditions^39, 40^. The RacRS two-component system is important for chicken colonisation and is part of a temperature-dependent signalling pathway^41^. In our study, Tn mutants in *racS* had reduced survival in chicken juice and tap water at low temperature. Chemotaxis has also been suggested to play a role in survival at low temperature^42^. In line with this, Tn mutants of *mcp4_2* were attenuated for survival in chicken juice and tap water. In addition, Tn mutants in *kefB* and *czcD* (antiporters), *fabI* (fatty acid metabolism) and CJM1cam_0181 to CJM1cam_0183 (*purN*, *nnr*, and a gene encoding a hypothetical protein) were linked to survival in both chicken juice and tap water.

**Figure 5:**
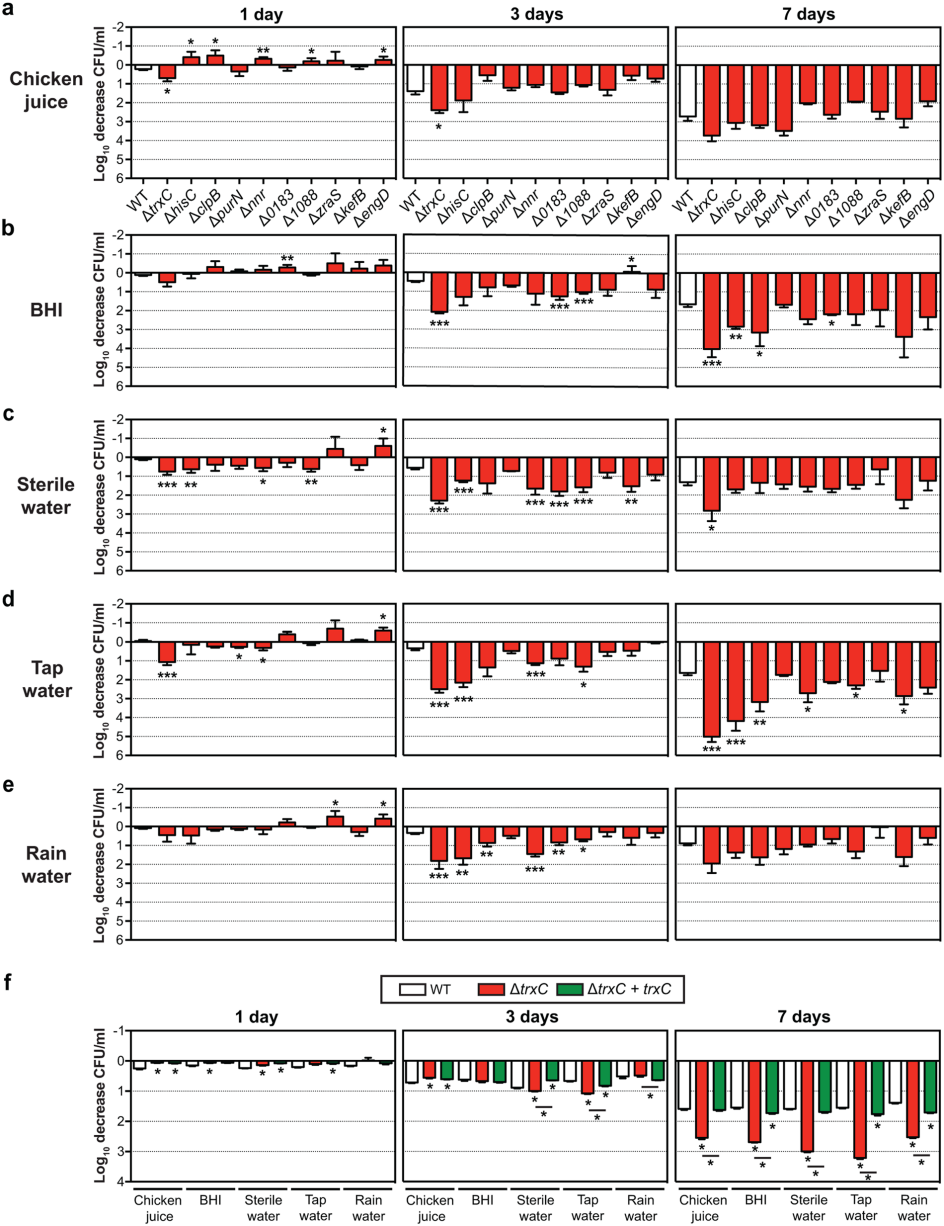
Validation of Tn mutant library screen during survival at low temperature under nutrient-rich and –poor conditions. (**a–e**) Survival of defined *C*. *jejuni* M1cam gene deletion mutant in (**a**) BHI, (**b**) chicken juice, (**c**) sterile water, (**d**) tap water, (**e**) rain water. Survival of *trxC* defined gene deletion mutant and complemented mutant (**F**), at 4 °C in different media. Data shown are Log_10_ decrease of CFU/ml relative to the 0 day time-point and plotted as means with SEM (*n* ≥ 4). Statistical significance was analysed using a Mann-Whitney test with *P < 0.05, **P < 0.01 and ***P < 0.001.

**Figure 6:**
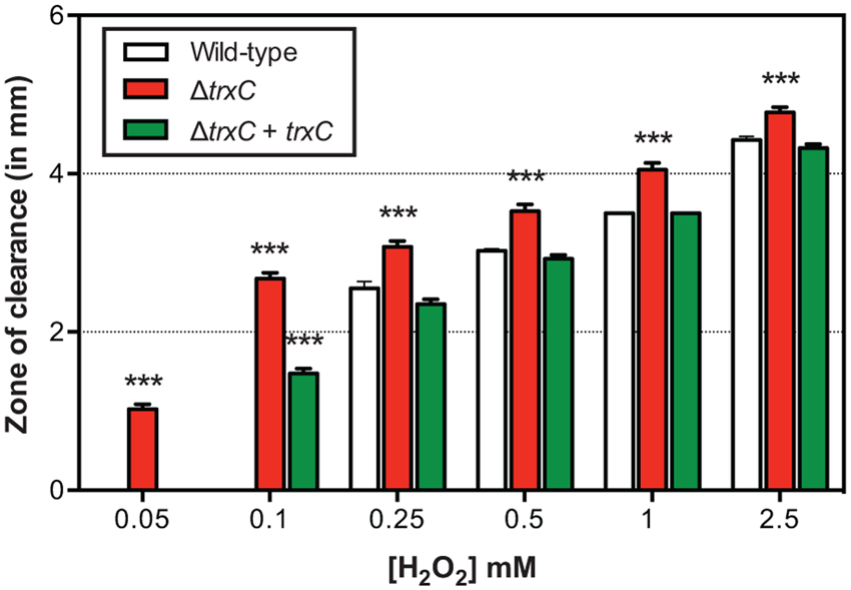
Sensitivity to hydrogen peroxide of *C*. *jejuni* M1cam wild-type, *trxC* defined gene deletion mutant and *trxC* complemented mutant. Data shown are zones of inhibition and plotted as means with SEM (*n* ≥ 4). Statistical significance was analysed using a two-way ANOVA test with ***P < 0.001.

For validation, 10 defined gene deletion mutants were assayed under all conditions described above. We found that several deletion mutants were attenuated for survival after 3 days in BHI and water (sterile, tap and rain). However, at 7 days their survival did not differ from the wild-type. This is most likely due to further decreasing levels of the wild-type between day 3 and 7 (Fig. 5a). Confirmatory experiments with deletion mutants highlighted the contribution of *hisC* (aromatic amino acid aminotransferase) and *trxC* (thiol-disulphide oxidoreductase) for survival at low temperature (Fig. 5b–e). The *hisC* mutant showed attenuated survival in BHI (7 days), sterile water (3 days), tap water (3 and 7 days) and rain water (3 days) (Fig. 5b–e). Deletion of *trxC*, however, resulted in reduced survival of *C*. *jejuni* in BHI, sterile water and tap water after 7 days (Fig. 5b–d) and in chicken juice and rain water after 3 days (Fig. 5a,e). Although the deletion of *trxC* resulted in a prolonged lag-phase during growth *in vitro* in BHI broth (Fig. S7), genetic complementation of the *trxC* deletion restored its phenotype under all conditions tested, confirming the importance of *trxC* in the survival of *C*. *jejuni* at 4 °C, both in nutrient-rich and –poor conditions (Fig. 5b). Thus far, the function of TrxC in *C*. *jejuni* is unknown. The closest ortholog is TrxC in *Helicobacter pylori*, which is required for protection against oxidative stress^43^. The M1cam *trxC* mutant showed increased sensitivity to hydrogen peroxide, which was restored to near wild type levels in the genetically complemented mutant (Fig. 6), supporting its role in the response to oxidative stress.

### Infection of human gut epithelial cells

The M1cam mutant library ‘C’ was used to infect Caco-2 human gut epithelial cells. To identify genes required for adhesion, Tn mutants that adhered to Caco-2 epithelial cells were compared to the non-adherent fraction rather than to the inoculum. This was to restrict the number of false positives due to attenuated survival in the infection medium. Comparing the Tn mutants that invaded the epithelial cells with the non-adherent fraction identified 57 candidate genes involved in cellular invasion, whereas only two genes passed our filtering criteria for adhesion (Fig. 2 and Tables S3 and S4).

In line with previous studies^3, 22, 44, 45^, genes linked to the COG class “cell motility” were overrepresented amongst the genes required for cellular invasion (Fig. 2 and Fig. S5). Gao *et al*., screened a Tn mutant library in *C*. *jejuni* 81–176 in a Cos-1 monkey kidney fibroblast cell model, which identified 36 invasion genes^22^, of which 19 genes were also identified in this study using Caco-2 human gut epithelial tissue culture cells. A total of 38 genes were only required for invasion by M1cam and 17 only for 81–176^22^. Of the M1cam unique invasion genes, 14 were linked to the flagellar system, including *fliK*, *flaG*, *fliD*, *fliW*, and *maf3*. The differences in invasion requirements are likely to be caused by the different cell-types or related to the *C*. *jejuni* strains.

**Figure 7:**
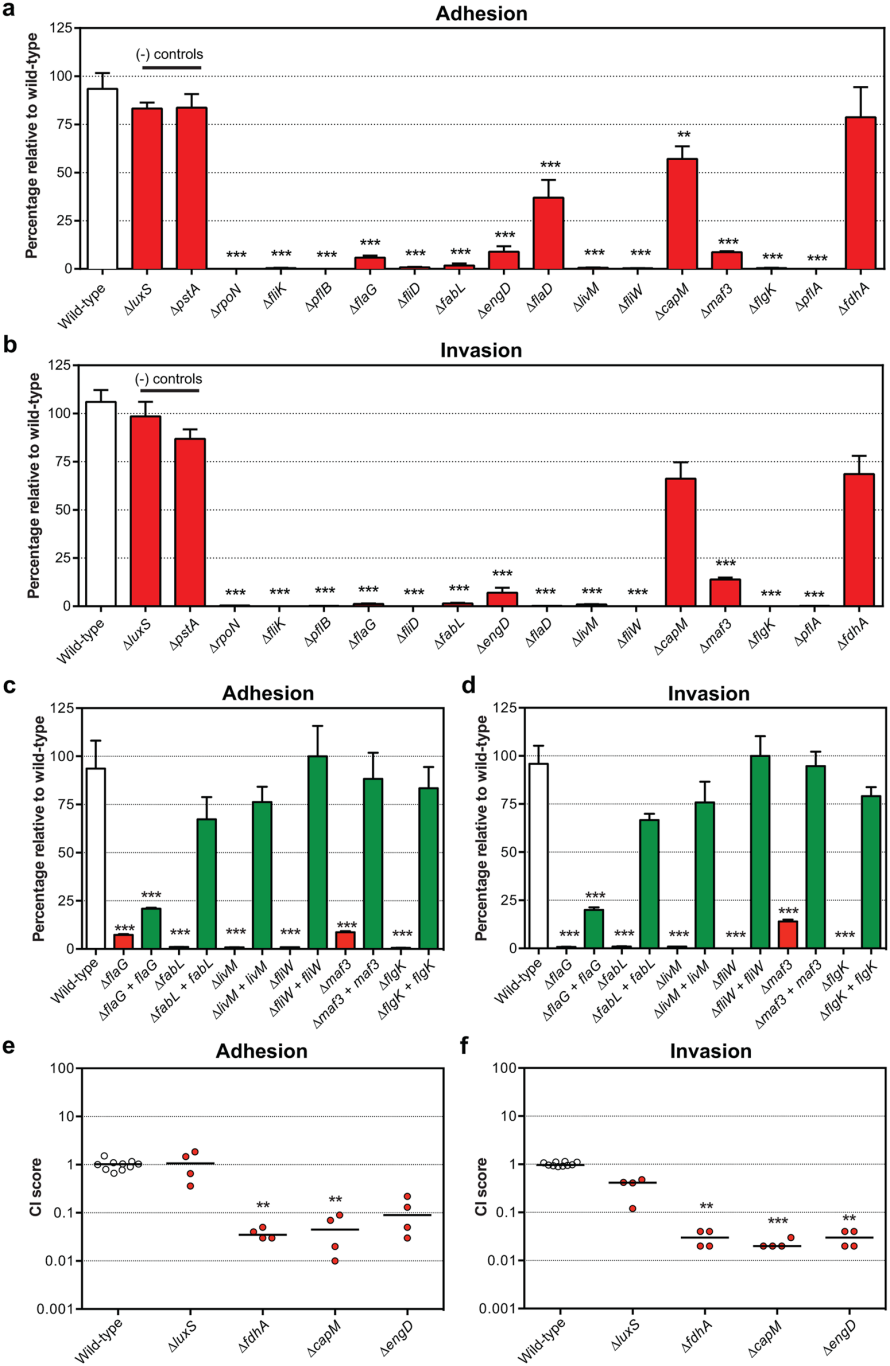
Validation of Tn mutant library screen during adhesion and invasion of human gut epithelial tissue culture cells. Adhesion to (**a**), and invasion (**b**) of, Caco-2 cells by *C*. *jejuni* M1cam defined gene deletion mutants. Mutants in *luxS* and *pstA*, which were not identified in our Tn-seq screen with Caco-2 cells, were included as negative controls and *rpoN*, which was previously shown to be required for adhesion and invasion^81, 82^, served as a positive control. Caco-2 adhesion (**c**), and invasion (**d**), of gene deletion and genetically complemented mutants. Data is represented as percentage of wild-type (*n* ≥ 3) and plotted as means and SEM. The competitive index (CI) was calculated by dividing the ratio of mutant to wild-type bacteria recovered upon (**e**) adhesion to, and (**f**) invasion of, Caco-2 cells by the ratio of mutant to wild-type bacteria that were used in the inoculum. Statistical significance was calculated using a Mann-Whitney test where *P < 0.05, **P < 0.01 and ***P < 0.001.

Validation was performed with 15 defined gene deletion mutants, this included genes not previously reported to be linked to motility or the flagellar system (Fig. 7a,b). This confirmed the role of 13 out of 15 selected invasion genes (Fig. 7b). Tn-seq only identified two genes involved in adhesion, however 14 out of 15 tested deletion mutants displayed attenuated adhesion (Fig. 7a). This apparent discrepancy might be due to differences in the experimental set-up between the Tn library screen and the validation experiments. It is well recognized that between Tn mutants is a confounding factor in Tn library screens^23, 45^, in contrast to the validation experiments presented here, in which defined gene deletion mutants were allowed to interact with Caco-2 cells as single mutant inocula. Further interactions between adhesion-deficient and –proficient Tn mutants may compensate for adhesion of otherwise adhesion-deficient Tn mutants. Amongst the genes that were confirmed to play a role in interacting with Caco-2 cells were *livM* (amino acid metabolism), *fabL* (fatty acid metabolism) and *engD* (GTP-dependent nucleic acid-binding protein) that have no known link to the flagellar system. However, deletion of *livM* resulted in ~50% reduced motility compared to the wild-type (Fig. S6). No genomic variations in the *livM* mutant linked to motility were identified (Table S5). The role of *engD* in colonisation of chickens was not confirmed in validation experiments with a defined deletion mutant (Fig. 3). However, we confirmed the contribution of *engD* in adhesion and invasion of Caco-2 tissue culture cells (Fig. 7). The *maf3* gene deletion mutant was motile (Fig. S6) but had a reduced capacity to colonise chickens (Fig. 3a,b) and also lacked the ability to adhere to and invade Caco-2 cells (Fig. 7a–d).

Genetic complementation of *fabL*, *livM*, *fliW*, *maf3* and *flgK* mutants restored their adhesion and invasion capacity to wild type-levels, confirming their role in the interaction with human gut epithelial cells (Fig. 7c,d). Genetic complementation of the *flaG* mutant partially restored its phenotype, which may be due to deregulated *fliD* or *fliS* expression as we observed for the *fliD* mutant (Fig. S8). Complementation of the *engD* mutant was unsuccessful due to the lack of expression of *engD* (Fig. S8). Further, the *capM* and *fdhA* Tn-seq phenotypes were confirmed when mutants were assayed in competition with the wild-type (Fig. 7e,f), however in mono-infection no attenuation was observed (Fig. 7a–d). The *luxS* negative control mutant was not attenuated for adhesion and invasion in competition with the wild-type (Fig. 7e,f).

## Discussion

Here we present a comprehensive analyses of gene fitness in *C*. *jejuni*. Our Tn-seq data identified 486 genes that are essential for fitness in *C*. *jejuni*. When compared with the literature, till date, mutants in 38 out of 486 genes have been reported. Although some of these mutants had a severe growth defect, such discrepancies could arise due to the differences in culture conditions or culture medium while generating transposon libraries, or the presence of competition with other Tn mutants (which is not the case during the generation of defined gene deletion mutants). Potential drawbacks of various experimental designs and how to improve Tn-seq based methods have been discussed in great detail in a recent review by Chao *et al*.^46^. Profiling the genes required for *in vitro* growth of three *C*. *jejuni* strains underscored that a large part of the genome (~27%) is vital to fitness (Table S2). Most likely as a consequence of a low redundancy in its relatively minimal genome and transcriptional coupling of genes^47, 48^. This implies that there could be opportunities for targeting some of these genes for novel intervention strategies, *e*.*g*. as previously reported by Mobegi *et al*.^49^.

**Figure 8:**
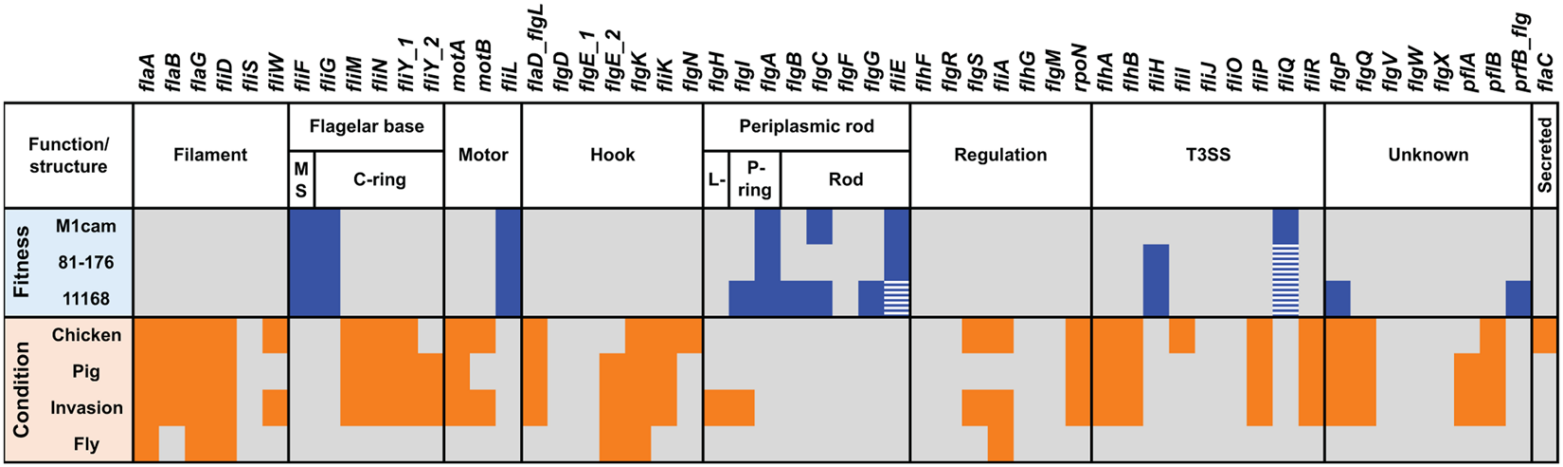
Overview of flagellar system genes required for fitness during *in vitro* growth and in model representing host interaction. Genes involved in the flagellar system that were required for fitness during *in vitro* growth of *C*. *jejuni* strains M1cam, 81–176 and 11168 are indicated in blue. Genes that passed the fitness score cut-off but did not pass the 0.95 probability for Tn inactivation are indicated with blue dashed boxes. Genes which in the Tn library screens were shown to be required for chicken colonisation, gnotobiotic piglet infection, invasion of human gut epithelial tissue culture cells or survival in houseflies are indicated in orange.

Elements of the flagellar system, *i*.*e*. the flagellar base and periplasmic rod structure and T3SS (Fig. 8), which when inactivated by a Tn have a severe impact on fitness, provide a potential focus for intervention along with genes required for the LOS lipid A and KDO moieties. We also found that inclusion of the first L-*glycero*-D-*manno*-heptose residue (catalyzed by WaaC) was required for fitness. These structural elements have a critical role during host interaction and therefore represent promising targets for developing intervention strategies^44, 50^. Gene fitness analysis revealed that components of the gluconeogenesis pathway required for biosynthesis of glucose-(derivatives) that serve as building blocks for both LOS and CPS as well as the *N*- and *O*-linked protein glycosylation pathways^51^, were essential for the growth of *C*. *jejuni*. In addition, the type II fatty acid synthesis pathway (FASII) genes *fabDFGHZ* were required for fitness. Interestingly, there are several classes of FASII pathway inhibitors with potent antimicrobial properties and consequently fatty acid synthesis is also considered a lucrative target for antibiotics^49, 52^.

The same *C*. *jejuni* M1cam Tn library was screened in all of the experimental models presented in this study and compared with data derived from screening the same Tn library during infection of gnotobiotic piglets (De Vries *et al*., submitted). Twenty-eight genes were found to be required in all three host interaction models (chicken, pig, and cellular invasion), of which 21 genes belong to the flagellar system. Amongst the genes that were required across three host models and that did not belong to the flagellar system were *engD*, *livM* and *capM* (Figs 3 and 7). The EngD ortholog in *E*. *coli* (YchF) belongs to the GTPase family and is a negative regulator of the oxidative stress response^53^. YchF proteins have been implicated in pathogenesis of other bacterial species^53, 54^. With branched-chain amino acids (BCAA) being linked to chemotaxis^55^, *livM*, part of the ABC-type BCAA transport system (*livM*, *livH*, *livK*, and *livJ*), may be implicated in within host chemotaxis. CapM/PglH plays a role in the glycan assembly process and adds the final *N*-acetlygalactosamine residues during surface decoration^56^. Previous studies demonstrated a role for the *N*-linked protein glycosylation gene *capM*/*pglH* in chicken colonisation^57, 58^, along with other members of the surface protein glycosylation locus (*pglBEF*)^59^. Our chicken colonization Tn-seq screen showed that Tn mutants in *pglB*, *pglF* and *pglI* had reduced colonisation (Table S3), whereas *pglACD* were required for fitness during *in vitro* growth (Table S2).

Ninety-six genes were only identified in the chicken colonisation screen and not during infection of gnotobiotic piglets, including phosphate metabolism genes (*pstABS* and *phoR*). PstA appears to be unrelated to motility (Fig. S6), deletion of *pstA* did not affect adhesion and invasion of human gut epithelial cells (Fig. 7), but was required for *C*. *jejuni* to colonise chickens (Fig. 3a,b). The phosphate regulon in *C*. *jejuni* 81–176 is increased in 1-day-old chicks, which suggests that phosphate levels might be low in chickens, consequently activating the phosphate regulon^35^. The phosphate regulon might operate in a second messenger system that (in)directly regulates expression of chicken colonisation genes^60^. However, it remains to be investigated which *C*. *jejuni* genes are under control of this system. Tn mutants in genes involved in chemotaxis, *mcp4*_1 and *cheV*, were uniquely attenuated during chicken colonisation, whereas Tn mutants in *mcp4*_3 and *mcp4*_4 were uniquely attenuated during gnotobiotic piglet infection, suggesting the existence of host- or substrate-specific chemotaxis. A large number of genes involved in the utilisation of amino acids and organic acids such as lactate, pyruvate, acetate and tricarboxylic acid (TCA) cycle intermediates only appear to be required during gnotobiotic piglet infection. These host specific requirements are most likely related to dietary differences between chickens and piglets. Our Tn library screens indicated that pyruvate kinase (*pyk*) is important in both chickens and piglets. Pyk is part of the Embden-Meyerhof-Parnas pathway, and shifting the metabolic flux at the level of Pyk has previously been suggested as a potential antimicrobial strategy^61^.

A detailed analysis of the flagellar system across experimental models indicated that the extracellular part of the flagellum, the filament and hook structure, plays a vital role in *C*. *jejuni* (Fig. 8). This included the filament chaperone gene *fliW* ^13, 62^ and the flagellar cap *fliD*. A FliD subunit vaccine induced a transient but significant reduction (~2 log) of *C*. *jejuni* in the chicken caecum^63^, demonstrating its potential as a target for intervention. Linked to the flagellar system is also the motility accessory factor 3 (*maf3*). We found that a *maf3* deletion mutant was motile, but displayed attenuated chicken colonisation and adhesion and invasion of Caco-2 cells. Although *maf3* is located in the flagellar glycosylation locus^64^, deletion of *maf3* in *C*. *jejuni* 81–176 did not result in an altered glycosylation of the flagellar filament^65^.

Experimental models of *C*. *jejuni* survival and transmission, *i*.*e*. in the housefly and under low temperature conditions, were less stringent than the *in vivo* and *in vitro* host-pathogen models, reflected by subtler fold-changes compared to the host interaction models. This results in a lower number of candidate genes being identified and a lower conformation in validation experiments with defined gene deletion mutants (Figs 4 and 5). Interestingly, this led to a novel potential target for intervention not linked to host interactions. The *trxC* gene was required for survival at low temperature, both in nutrient-rich and –poor conditions (Fig. 5) and for resistance against peroxide (Fig. 6), indicating a role in the oxidative stress response.

*C*. *jejuni* is considered the leading cause of bacterial gastroenteritis, however our understanding of its biology is limited. The development of future intervention strategies might best be aided by a thorough understanding of the biology of *C*. *jejuni* in its life cycle. Our work indicates that many genes/pathways make indispensable contributions to the ability of *C*. *jejuni* to thrive in the host and environment. We anticipate that the findings from this study and the use of the Tn mutant libraries in future studies will provide a continued insight into the mechanisms required for growth as well as survival within and outside host organisms. Most importantly, our comprehensive screening approach has again clearly shown that the flagella drive *C*. *jejuni* interaction with its hosts. Therefore, future efforts should focus on how to exploit this to effectively control infections caused by *C*. *jejuni*.

## Methods

### Bacterial strains and growth conditions

Wild-type strains, defined gene deletion mutants, genetically complemented mutants and plasmids are summarised in Table S6. *C*. *jejuni* strains M1cam (derivative of M1 used in our laboratory)^13, 14^, 11168^10^, and 81–176^15^ were cultured in Brain Heart Infusion (BHI) broth or on BHI agar supplemented with 5% (v/v) defibrinated horse blood in the presence of 5 µg/ml trimethoprim (TrM). Tn mutant libraries and defined gene deletion mutants were grown in the presence of 10 µg/ml chloramphenicol (Cm) and 50 µg/ml kanamycin (Km) was added for culturing genetically complemented mutants. *C*. *jejuni* were grown under microaerophilic conditions (5% O_2_, 10% CO_2_, 85% N_2_) in a MACS VA500 Variable Atmosphere Work Station (Don Whitley, Shipley, United Kingdom). Liquid cultures were grown with agitation (200 rpm). For use in the experimental models, *C*. *jejuni* M1cam wild-type, defined gene deletion mutants and genetically complemented mutants were cultured for ~48 h, re-plated on fresh plates and grown for another 16 h. Tn mutant libraries were grown from freezer stocks on 9 × 90 mm BHI blood agar plates (200 μl per plate) and grown for 16 h. *E*. *coli* NEB 5α or 10-β (New England Biolabs) were used for cloning and were cultured in Luria Bertani (LB) medium with appropriate antibiotics, at 37 °C.

### Construction of defined gene deletion mutants and genetically complemented mutants

*C*. *jejuni* M1cam gene deletion mutants were constructed by allelic replacement of the gene with a chloramphenicol (*cat*) resistance cassette as described in de Vries *et al*.^13^. Gene deletion mutants were subjected to phenotypic and genotypic characterisation, including motility (Fig. S6) and *in vitro* growth in liquid culture (Fig. S7). Whole genome sequencing (WGS) based variant analysis (single nucleotide polymorphisms [SNP] and insertion/deletions [INDELS]) of defined deletion mutants was used to screen for second-site mutations that might have affected the phenotypes under investigation. Variants were detected at 129 positions relative to the *C*. *jejuni* M1cam reference genome^13^ (Table S5). The variant database was cross-referenced with data obtained in Tn mutant library screens to assess whether the gene affected by the variant had a potential impact on the phenotype under investigation. In addition, phenotypes of deletion mutants sharing a variant were compared to predict possible confounding effects of the second-site mutation. This in-depth analysis did not identify confounding effects of second-site mutations.

Genetic complementation of *mcp4*_1, *fliK*, *flaG*, Δ*fliD*, *pstA*, *fabL*, *engD*, *livM*, *trxC*, *capM*, *maf3* and *flgK* mutants was performed using the pSV009 plasmid as described in de Vries *et al*.^13^ (Fig. S8). Predicted promoter region(s), including a ribosome-binding site (RBS) were derived from the Dugar *et al*. dataset^47^. For genes arranged in an operon, the promoter region was mostly identified upstream of the 5′ end of the transcript. These promoter regions were synthesised as a DNA string (GeneArt, Life Technologies, UK) and flanked with appropriate restriction sites. The *fliK*, *fliD*, *pstA*, *engD* and *maf3* open reading frames (ORFs) were cloned into *Xba*I and *BamH*I sites in pSV009 and promoter regions were inserted in the *Xho*I and *Xba*I sites, resulting in the promoter-gene fusions. For *mcp4*_1, *flaG*, *fabL*, *trxC*, *capM* and *flgK*, a promoter consensus region was identified upstream of the start codon. A fragment containing the ORF plus ~200 bp upstream was cloned in the *Xho*I and *BamH*I sites of the plasmid pSV009. As no obvious promoter was identified for *livM*, its transcription was placed under the control of a *cat* promoter by cloning the ORF into *Xba*I and *BamH*I sites of pSV009. Genetic complementation fragments were amplified from the pSV009-derived plasmids using primers pSV009_GCampl _FW1/RV1. The genetic complementation fragments were introduced into respective M1cam defined gene deletion mutants by electroporation as described in de Vries *et al*.^13^. Oligonucleotide sequences are listed in Table S7.

### Gene expression analysis in genetically complemented gene deletion mutants

Total RNA was isolated as previously described in de Vries *et al*.^66^. DNA-free total RNA (500 ng) was reverse transcribed into cDNA using the QuantiTect reverse transcription kit (Qiagen). Real-time quantitative PCR was performed using the SensiFast SYBR No-ROX Kit (Bioline) on a Rotor-Gene Q machine (Qiagen). Gene expression fold-changes in genetically complemented mutants were calculated relative to wild-type using the ΔΔCt method^67^ with *gyrA* as a reference (two biological replicates with two technical replicates).

### Growth kinetics

*C*. *jejuni* wild-type and gene deletion mutants were harvested from BHI blood agar plates in BHI broth. Culture suspensions were diluted to OD_600nm_ ~ 0.2 and used to inoculate 5 ml pre-warmed BHI broth at an OD_600nm_ of ~0.005. Growth was monitored after 4, 8 and 24 h incubation at 200 rpm at 42 °C under microaerophilic conditions (*n* ≥ 3), by optical density and by plating ten-fold serial dilutions.

### Analysis of motility

Motility assays (*n* = 3) were performed in semi-solid agar as described in de Vries *et al*.^13^.

### Genome sequencing of defined gene deletion mutants

Sequencing libraries were prepared using the NEBNext Ultra or Ultra II DNA library prep kit (New England Biolabs) and sequenced on the MiSeq platform as described in de Vries *et al*.^13^. Reads were mapped to the M1cam reference genome (accession no. CP012149^13^) using Stampy^68^, variants were identified with Samtools^69^ and the effect at the protein level was predicted using SnpEff^70^. Deletion mutants in *pflA* and *flaD* were analysed previously^13^.

### Construction of Tn mutant libraries in *C*. *jejuni*

A Tn donor plasmid suitable for Tn-seq^16^ was constructed by amplifying the *mariner* Tn encoding the Cm-resistance cassette from pAJG39^29^ using a single 5′-phosphorylated primer PBGSF20, introducing *Mme*I restriction sites within the inverted repeats of the Tn element. The Tn element was sub-cloned into pJET1.2 (Thermo Scientific) and the resulting plasmid pSV006, was used for *in vitro* Tn mutagenesis as described in Holt *et al*.^71^ with minor adjustments. Briefly, 2 μg of *C*. *jejuni* DNA was incubated for 5 h at 30 °C with 1 µg of pSV006 and ~250 ng Himar1-C9 transposase, which was purified as described in Akerley *et al*.^72^. After end-repair with T4 DNA polymerase and *E*. *coli* DNA ligase the mutagenesised DNA was transferred to *C*. *jejuni* by natural transformation^13^. Tn transformants were harvested from plates and pooled. The pooled library was used to inoculate 100–200 ml BHI-TrM-Cm broth to an OD_600nm_ of ~0.1 and grown overnight), yielding “working stocks”. Genomic DNA was isolated with Genomic-tip columns (Qiagen).

### Gene fitness analysis

Tn insertion sites were identified using Tn-seq^16^, as described in Burghout *et al*.^73^ with minor adjustments. Briefly, Tn mutant library DNA was fragmented by *Mme*I restriction digestion, adapters containing inline barcodes were ligated to the *Mme*I fragments, and amplified using the primers PBGSF29 and PBGSF30 with NEBNext high fidelity polymerase (New England Biolabs). Tn insertion sites were sequenced using single-end 40–50 bp sequencing on the Illumina HiSeq 2500. Sequence reads were demultiplexed using the FastX toolkit barcode splitter and analysed further with the ESSENTIALS pipeline^19^. Sequence reads were aligned to the *C*. *jejuni* genomes^13, 15, 74^ with a match of ≥16 nt. Kernel density plots were generated in R to distinguish “true” Tn insertions from “noise” sequencing reads, yielding a read count cut-off per Tn library. Insertion “hot spotting” was analysed by plotting the Log_2_ read count per chromosomal position using an in-house Perl script. As a measure of gene fitness, the Log_2_ fold-change of observed *vs* expected reads was calculated per gene, with Kernel density plots allowing accurate delineation of fitness (required for *in vitro* growth) and non-fitness genes^19^. Additional criteria were: a Benjamini & Hochberg adjusted *P* < 0.05 and a probability that the gene was inactivated by a Tn insertion of >0.95, as calculated using a derivative of Poisson’s law; 1 − e^N x Ln(1−f)^, with N = number of unique Tn insertion mutants and f being the gene size divided by the size of the genome. In addition, genes for which no sequence reads were detected and the probability of inactivation was >0.95 were considered to be required for fitness. For functional class enrichment analysis COGs were assigned to M1cam, 11168 and 81–176 proteins and consensus COGs were assigned to homologous groups (HGs; see next section). The overrepresentation of COG classes was assessed using a Fisher exact test with *Q*-value multiple testing correction^75^.

### Identification of homologs in *C*. *jejuni* strains

Protein sequences of *C*. *jejuni* M1cam, 11168 and 81–176 were clustered into putative HGs with OrthAgogue^76^. For this, a collective database was generated and proteins of each strain were queried against this database. The reciprocal best-hit protein pairs were identified by applying an e-value filter cut-off of 1e-5 to the “all against all” BLAST output (only proteins >50 amino acids in length were included). The putative HGs were identified by clustering with MCL with an inflation parameter of 2.6^77^.

### Chicken colonisation experiments

All chicken infection work was conducted in accordance with UK legislation governing experimental animals under project licence 40/3652 and was approved by the University of Liverpool ethical review process prior to the award of the licence. One-day-old Ross 308 broiler chicks were obtained from a commercial hatchery. Chicks were housed in the University of Liverpool, High Biosecurity Poultry unit. Chicks were individually tagged with leg rings or wing bands and maintained in floor pens at UK legislation recommended stocking levels allowing a floor space of 2,000 cm^2^ per bird at 25 °C on wood-shavings litter that was changed weekly prior to inoculation, and were given *ad libitum* access to water and a pelleted laboratory grade vegetable protein-based diet (SDS). Prior to experimental infection, all birds were confirmed as *Campylobacter*-free by taking cloacal swabs, which were streaked onto selective blood-free agar (mCCDA) (Lab M) supplemented with *Campylobacter* Enrichment Supplement (SV59) and grown for 48 h at 42 °C under microaerophilic conditions.

At 21 days of age, birds were inoculated by oral gavage with ~1.8 × 10^8^ CFU *C*. *jejuni* M1cam Tn library ‘C’. The birds were split into five cages (*n* = 6, 7, 7, 6, 6; group 1–5, respectively). Six days post-inoculation (p.i.), birds were killed by cervical dislocation. At necroscopy the caeca were removed aseptically and caecal content was collected. Next, the caecal contents were diluted 5- and 50-fold with Maximum Recovery Diluent (MRD) and 0.5 ml was plated per mCCDA-Cm (10 plates in total per dilution) for recovery of Tn mutants that successfully colonised the birds. Ten-fold dilution series were plated on mCCDA-Cm for enumeration. After 2 days growth, *C*. *jejuni* Tn mutants were recovered from the plates by scraping in 2 ml MDR, pelleted by centrifugation and stored at −80 °C. Genomic DNA was isolated from plate harvest pellets with Genomic-tip columns (Qiagen).

The composition of the input Tn mutant library (*n* = 3) and the library recovered per cage of birds (*n* = 4, cage 1–4), a cage was considered as a single unit of colonisation, was analysed by Tn-seq^16^. For this, 1 μg of DNA was pooled per group of birds (*n* = 6, 7, 7, 5 for groups 1 to 4, respectively). One bird from group 4 and 2 birds from group 5 could not be analysed due to heavy contamination on the recovery plates. As a result of this group 5 was eliminated from further analysis.

For validation with *C*. *jejuni* M1cam defined gene deletion mutants and genetically complemented mutants, birds were inoculated with ~1.8 × 10^8^ CFU, at 6 days p.i. chickens were killed by cervical dislocation, the caeca were removed aseptically and the caecal contents plates onto mCCDA plates for enumeration.

### Infection of houseflies

Newly pupated female houseflies (*Musca domestica*) were inoculated with 1 μl *C*. *jejuni* suspension *via* their proboscis^78^. For screening of Tn mutant survival, 5 groups of 10 flies were inoculated with ~10^6^ CFU M1cam Tn library ‘C’ on four different days. The flies were incubated for 4 h at 20 °C (in the dark) after which flies were homogenized using a Drigalsky spatula and 5 ml BHI was added. Large debris was removed through a low speed (700 × *g*) spin and the supernatant was adjusted to 5 ml with BHI before plating 0.5 ml per mCCDA-Cm (10 plates in total). Ten-fold dilution series were plated on mCCDA-Cm for enumeration. After 24 h growth, Tn mutants were recovered from plates by scraping in 2 ml BHI medium, centrifuged and pellets were stored at −80 °C for DNA isolation with Genomic-tip columns (Qiagen). Chromosomal DNA from the 5 groups of 10 flies per day was pooled; resulting in a single pooled sample for each 4 replicate. The composition of the input Tn mutant library (*n* = 4) and the library recovered per group of flies (*n* = 4) was analysed by Tn-seq^16^.

For validation experiments with *C*. *jejuni* M1cam gene deletion mutants (*n* ≥ 4) and genetically complemented mutants (*n* = 6), groups of 5 flies were inoculated with ~10^6^ CFU, killed 4 h p.i., and the bacterial load was quantified from pools of five flies or five flies individually (only for 2 replicates with gene deletion mutants), in both cases the average CFU per fly was used to calculate the Log_10_ decrease of CFU per fly relative to the inoculum.

### Cold survival assay

A suspension was prepared from *C*. *jejuni* M1cam Tn mutant library ‘C’ plate harvests, to an OD_600nm_ ~ 0.5 (~5 × 10^8^ CFU/ml). The Tn mutant library suspension was harvested by centrifugation and resuspended in either BHI or sterile tissue-culture grade water (experiment 1) or chicken juice, tap water or rain water (experiment 2). The chicken juice was prepared as follows: 10 frozen whole chickens were purchased from a commercial supplier and were allowed to defrost within the packaging for at least 16 h, as described by Brown *et al*.^79^. Concentrated liquid was recovered (>200 ml), sterilised through a 0.2 μM filter and stored at −20 °C. Tap water was obtained from a mains-fed tap in our laboratory that was allowed to run for at least 2 min prior to collecting the water used in experiments. To collect rain water, a large tub was left outside our laboratory overnight on a rainy evening. The rain water and tap water were passed through a 0.22 µM filter unit and stored at 4 °C. Chicken juice, tap water and rain water were screened for the presence of *Campylobacter* spp. by plating on mCCDA plates. The Tn library samples were incubated at 4 °C and aliquots were taken at 0 h, 6 h, 1 and 7 days for experiment 1 (*n* = 3) or at 1, 3, and 7 days for experiment 2 (*n* = 4) and plated for recovery of Tn mutants. Ten-fold dilution series were plated on BHI-TrM-Cm for enumeration. After 2 days incubation, Tn mutants were recovered from plates by scraping in 2 ml BHI per plate, centrifuged and pellets were stored at −80 °C for DNA isolation with Genomic-tip columns (Qiagen). The Tn library composition at time = 0 h was compared to the library recovered after incubation at 4 °C under the above mentioned conditions by Tn-seq^16^.

For validation experiments, the survival of gene deletion mutants and genetically complemented mutants was assayed as described above (*n* = 4).

### Infection of human gut epithelial cells

Caco-2 cells (ATCC CC-L244 HTB-37), were cultured in DMEM (Life Technologies) supplemented with 10% (v/v) heat inactivated FBS (Gibco), and 1% (v/v) non-essential amino acids (Sigma Aldrich), at 37 °C with 5% CO_2_. The *C*. *jejuni* M1cam Tn mutant library ‘C’ was used to infect seven 143 cm^2^ dishes per replicate (*n* = 4) containing a monolayer of Caco-2 cells at a multiplicity of infection (MOI) of 100 in low phosphate HEPES buffer (10 mM HEPES, 5.4 mM KCl, 145 mM NaCl, 5 mM glucose, 1 mM MgCl_2_, 1 mM CaCl_2_, 2 mM phosphate buffer pH 7.4)^80^. Cells were incubated for 2 h after which the non-adherent fraction from two dishes was recovered onto BHI blood agar plates. For adherence, two dishes were washed three times in Dulbecco’s PBS (D-PBS), Caco-2 cells were lysed in 10% (v/v) Triton-X100 solution in D-PBS, and bacteria were recovered on BHI blood agar plates. The remaining five dishes were washed three times in D-PBS and incubated for an additional 2 h in DMEM with 250 µg/ml gentamycin. Tn mutants that invaded Caco-2 cells were recovered after washing three times in D-PBS and Caco-2 cell lysis in 10% (v/v) Triton-X100 in D-PBS. After two days of growth on plates, Tn mutants were recovered from the plates, centrifuged and pellets were stored at −80 °C. DNA was isolated from harvested pellets using Genomic-tip columns (Qiagen).

Validation of the screen was performed using 24-well plates, for which Caco-2 cells were infected with *C*. *jejuni* M1cam defined gene deletion mutants and genetically complemented strains (*n* ≥ 4) as described above. Bacteria recovered from different fractions were serially diluted and plated on BHI agar plates containing appropriate antibiotics. Adhesion and invasion of Caco-2 cells by various *C*. *jejuni* strains was calculated relative to the matched wild-type (*n* ≥ 3). To test the effect of competition, Caco-2 cells were infected at an MOI of 100 with a mix of the wild-type strain to a defined gene deletion mutant at a ratio of 100:1. A competitive index (CI) score was calculated by dividing the ratio of mutant to wild-type recovered from adherent and invaded fractions by the ratio of mutant to wild-type bacteria in the inoculum (*n* ≥ 4).

### Conditionally essential gene analyses

Tn-seq data from the different experimental models (conditionally essential genes screens) was processed as described in “gene fitness analysis”. To identify genes of which Tn mutants were attenuated or enriched in the tested models, read counts were collected per gene and compared between output (recovered) and the input or control conditions (as defined above). Only genes covered by >100 reads in the control condition were considered, allowing assessment of 809 ± 58 (67 ± 5%) non-fitness genes (define ‘non-fitness genes’) in the selected models. The following filter steps were applied: a Log_2_ fold-change (FC) below the attenuated cut-off value or higher than the enriched cut-off value, Benjamini & Hochberg false discovery rate <0.05, and two or more Tn mutants showing a Log_2_ fold-change below the attenuated cut-off value or higher than the enriched cut-off value (analysed using a custom Python script). The Log_2_ fold-change cut-offs were selected based on MA-plots. In addition, 514 genes that were obligate essential or required for fitness (defined above) were eliminated from the analysis, see “gene fitness analysis”. COG functional class enrichment was analysed using a Fisher exact test with *Q*-value multiple testing correction^75^.

### Hydrogen peroxide sensitivity assay

*C*. *jejuni* strains were added to pre-cooled BHI agar (~45 °C) to a calculated OD_600nm_ ~0.005 and 25 ml of this media-bacterial suspension was poured into 90 mm petri dishes. Blank filter paper discs, 6 mm, were loaded with 10 µl of 0.05, 0.1, 0.25, 0.5, 1, 2.5, 5 or 7.5 M H_2_O_2_ solution. The discs were allowed to air dry and were then placed in the center of the solidified agar plates. The plates were incubated for 24 h, after which time the inhibition zone diameter was measured using a ruler (*n* = 4).

### Nucleotide sequence accession numbers

Tn-seq and genome sequencing data has been deposited in the European Nucleotide Archive (http://www.ebi.ac.uk/ena) and are available *via* study accession numbers PRJEB18797 and PRJEB18765, respectively.

### Statistical analysis

Statistical analysis was performed in GraphPad Prism v6.

## Electronic supplementary material

Supplementary Information

Table S2

Table S3

Table S4

Table S5
