# Supplementary material for: Genome-wide fitness analyses of the foodborne pathogen Campylobacter jejuni in in vitro and in vivo models
Source: Sci Rep. 2017 Apr 28;7:1251. doi: 10.1038/s41598-017-01133-4 (PMC5430854; doi:10.1038/s41598-017-01133-4)
Supplement: Supplementary file 1 — Supplementary Information [file 41598_2017_1133_MOESM1_ESM.pdf]

Supplementary information

Genome-wide fitness analyses of the foodborne pathogen *Campylobacter jejuni* in *in vitro* and *in vivo* models

Stefan P.W. de Vries[1§], Srishti Gupta[1§], Abiyad Baig[1a], Elli Wright[2], Amy Wedley[2], Annette Nygaard Jensen[3], Lizeth LaCharme Lora[2], Suzanne Humphrey[2b], Henrik Skovgård[4], Kareen Macleod[5], Elsa Pont[1], Dominika P Wolanska[1], Joanna L'Heureux[1], Fredrick M. Mobegi[6c], David G.E. Smith[7], Paul Everest[5], Aldert Zomer[8], Nicola Williams[9], Paul Wigley[2], Thomas Humphrey[10], Duncan J Maskell[1], Andrew J Grant[1]*

[1] Department of Veterinary Medicine, University of Cambridge, Cambridge, United Kingdom.

[2] Department of Infection Biology, Institute of Infection and Global Health, University of Liverpool, Leahurst Campus, Neston, United Kingdom.

[3] Technical University of Denmark, National Food Institute, Copenhagen, Denmark

[4] Department of Agroecology, University of Aarhus, Slagelse, Denmark.

[5] University of Glasgow, Veterinary School, Glasgow, United Kingdom.

[6] Department of Paediatric Infectious Diseases, Radboud Institute for Molecular Life Sciences, Radboud University Medical Centre, Nijmegen, the Netherlands

[7] Heriot-Watt University, School of Life Sciences, Edinburgh, Scotland, United Kingdom.

[8] Department of Infectious Diseases and Immunology, Faculty of Veterinary Medicine, Utrecht University, Utrecht, The Netherlands.

[9] Department of Epidemiology and Population Health, Institute of Infection and Global Health, University of Liverpool, Leahurst Campus, Neston, United Kingdom.

[10] School of Medicine, Institute of Life Sciences, Swansea University, Swansea, United Kingdom.

[a] Current address: School of Veterinary Medicine and Science, University of Nottingham, Sutton Bonnington, Leicestershire, United Kingdom.

[b] Current address: Institute of Infection, Immunity and Inflammation, University of Glasgow, Glasgow, United Kingdom

[c] Current address: Division of Molecular Carcinogenesis, The Netherlands Cancer Institute, Amsterdam, The Netherlands

* Address correspondence to Andrew J Grant: E-mail ajg60@cam.ac.uk

[§] S.P.W.d.V and S.G. contributed equally to this work.

Strain	DNA	Library	Log_2 read cut-off [a]	Tn insertions in library	Unique Tn insertions per isolate
M1cam	Genome	A	6.31	3,277	23,334
	Genome	B	3.92	3,780	
	Genome	C	3.02	9,951[b]	
	Genome	D	4.30	11,745	
11168	Genome	A	3.44	6,261	15,008
	Genome	B	2.12	10,185	
81-176	Genome	A	4.60	4,395	17,827
		B	2.60	11,562	
		C	3.81	4,724	
	pVir	A	4.90	339	2,007
		B	2.52	939	
		C	3.84	1,530	
	pTet	A	4.51	341	1,919
		B	2.18	1,070	
		C	3.79	1,221	

[a] read cut-off was determined using density plots in R to detect minimum between background reads and reads derived from "true" Tn insertions (see Methods).
[b] M1cam library 'C' was selected for our conditionally essential gene analysis, as it had the highest number of genes (1,124) harbouring Tn insertions (M1cam libraries 'A', 'B' and 'D' had 909, 804 and 1,071 genes inactivated by Tn insertions, respectively).

Table S1 Overview of Tn mutant libraries constructed in *C. jejuni* M1cam, 11168, and 81-176.

Table S2 Overview gene fitness analysis in *C. jejuni* M1, 11168 and 81-176. As a measure of gene fitness, the Log_2 fold-change of observed *vs* expected reads was calculated per gene, with Kernel density plots allowing accurate delineation of fitness and non-fitness genes in each *C. jejuni* strain. Additional selection criteria were: a Benjamini & Hochberg adjusted $P < 0.05$ and a probability that the gene was inactivated by a Tn insertion of > 0.95, as calculated using a derivative of Poisson's law. In addition, genes for which no sequence reads were detected and the probability of inactivation was > 0.95 were included. For comparative analysis of fitness genes in the three *C. jejuni* strains, homologs were identified; see Materials and Methods for a detailed description.

Table S3 Overview of conditionally essential gene analysis. To identify genes of which Tn mutants were attenuated or enriched in the tested experimental models, read counts were collected per gene and compared between output (recovered) and the input or control conditions. The following filter steps were applied: genes represented > 100 reads in the input/control condition, a Log_2 fold-change (FC) below the attenuated cut-off value or higher than the enriched cut-off value, Benjamini & Hochberg false discovery rate < 0.05, and two or more Tn mutants showing a Log_2 fold-change below the attenuated cut-off value or higher than the enriched cut-off value. The Log2 fold-change cut-offs were selected based on MA-plots. In addition, the 514 genes that were obligate essential or required for fitness in *C. jejuni* M1cam (TABLE S2) were eliminated from this analysis.

Table S4 Conditionally essential genes grouped per functional (COG) category. Effect of Tn insertions on the ability of *C. jejun*i M1cam to colonize commercial broiler chickens, infect gnotobiotic piglets, adhere and invade human gut epithelial tissue culture cells, survive in houseflies and at 4°C in various media (chicken juice, BHI, tap water, rain water, and sterile water). Genes of which Tn mutants showed significantly attenuated or enriched fitness in the tested experimental models are listed and are grouped according to their COG functional classification. Data represented as Log_2 fold-change is also presented in FIG 2a. Orange = significantly attenuated, Blue = significantly enriched, and Grey = no significant Tn-seq Log_2 fold-change.

Table S5 Whole genome sequencing (WGS) based variant analysis of *C. jejuni*

Strain or plasmid	Relevant genotype or description	Source
***C. jejuni* strains**		
M1cam	Wild-type; derivative of M1	[1,2]
11168	Wild-type	[3]
81-176	Wild-type	[4]
**M1cam derivatives**		
Δ*mcp4*_1	ΔCJM1cam_0017; Cm^r	This study
Δ*mcp4*_1 + *mcp4*_1	ΔCJM1cam_0017 + CJM1cam_0017; Cm^r Km^r	This study
Δ*fliK*	ΔCJM1cam_0049; Cm^r	This study
Δ*moaA*	ΔCJM1cam_0163; Cm^r	This study
Δ*purN*	ΔCJM1cam_0181; Cm^r	This study
Δ*nnr*	ΔCJM1cam_0182; Cm^r	This study
ΔCJM1cam_0183	ΔCJM1cam_0183; Cm^r	This study
Δ*eptA*	ΔCJM1cam_0239; Cm^r	This study
Δ*hisC*	ΔCJM1cam_0297; Cm^r	This study
ΔCJM1cam_0303	ΔCJM1cam_0303; Cm^r	This study
Δ*pflB*	ΔCJM1cam_0368; Cm^r	This study
ΔCJM1cam_0438	ΔCJM1cam_0438; Cm^r	This study
Δ*glnP*	ΔCJM1cam_0449; Cm^r	This study
Δ*clpB*	ΔCJM1cam_0487; Cm^r	This study
Δ*flaG*	ΔCJM1cam_0522; Cm^r	This study
Δ*flaG* + *flaG*	ΔCJM1cam_0522 + CJM1cam_0522; Cm^r Km^r	This study
Δ*fliD*	ΔCJM1cam_0523; Cm^r	This study
Δ*ilvB*	ΔCJM1cam_0551; Cm^r	This study
Δ*pstA*	ΔCJM1cam_0593; Cm^r	This study
Δ*pstA* + *pstA*	ΔCJM1cam_0593 + CJM1cam_0593; Cm^r Km^r	This study
Δ*rpoN*	ΔCJM1cam_0642; Cm^r	This study
Δ*fabL*	ΔCJM1cam_0784; Cm^r	This study
Δ*fabL* + *fabL*	ΔCJM1cam_0784 + CJM1cam_0784; Cm^r Km^r	This study
Δ*flaD*	ΔCJM1cam_0851; Cm^r	[1]
Δ*engD*	ΔCJM1cam_0894; Cm^r	This study
Δ*jlpA*	ΔCJM1cam_0958; Cm^r	This study
Δ*livM*	ΔCJM1cam_0990; Cm^r	This study
Δ*livM* + *livM*	ΔCJM1cam_0990 + CJM1cam_0990; Cm^r Km^r	This study
Δ*fliW*	ΔCJM1cam_1052; Cm^r	[1]
Δ*fliW* + *fliW*	ΔCJM1cam_1052 + CJM1cam_1052; Cm^r	[1]
Δ*trxC*	ΔCJM1cam_1083; Cm^r	This study
Δ*trxC* + *trxC*	ΔCJM1cam_1083 + CJM1cam_1083; Cm^r Km^r	This study
ΔCJM1cam_1088	ΔCJM1cam_1088; Cm^r	This study
Δ*capM*	ΔCJM1cam_1107; Cm^r	This study
Δ*capM* + *capM*	ΔCJM1cam_1107 + CJM1cam_1107; Cm^r Km^r	This study
ΔCJM1cam_1125	ΔCJM1cam_1125; Cm^r	This study

Δ*luxS*	ΔCJM1cam_1180; Cm^r	This study
Δ*kefB*	ΔCJM1cam_1213; Cm^r	This study
Δ*maf3*	ΔCJM1cam_1291; Cm^r	This study
Δ*maf3* + *maf3*	ΔCJM1cam_1291 + CJM1cam_1291; Cm^r Km^r	This study
Δ*flgK*	ΔCJM1cam_1412; Cm^r	This study
Δ*flgK* + *flgK*	ΔCJM1cam_1412 + CJM1cam_1412; Cm^r Km^r	This study
Δ*zraS*	ΔCJM1cam_1436; Cm^r	This study
Δ*fdhA*	ΔCJM1cam_1456; Cm^r	This study
Δ*pflA*	ΔCJM1cam_1501; Cm^r	[1]
Δ*gltA*	ΔCJM1cam_1625; Cm^r	This study
***E. coli* strains**		
NEB 10β	Host strain for pSV009 and derivatives thereof	New England Biolabs
NEB 5α	Host strain for pSV009 and derivatives thereof	New England Biolabs
DH5α	Host strain for pSV006	Life Technologies
BL21 pLysS	Host strain for expression of *Himar1* transposase	Novogen
**Plasmids**		
pAJG39	Donor *mariner* Tn element encoding Cm^r	[5]
pSV006	*Mariner* Tn (Cm^r) donor plasmid used for *in vitro* Tn mutagenesis; pJET1.2 backbone (Fermentas)	
pMALC9	Plasmid for expression *Himar1* transposase fused to the maltose-binding protein (MBP); pMal-cri backbone (New England Biolabs)	[6]
pCC027	Cm^r source for generation of defined gene deletion mutants in *C. jejuni*	[7]
pMiniT	*E. coli* PCR cloning plasmid	New England Biolabs
pUC19	*E. coli* cloning plasmid	New England Biolabs
pSV009	*C. jejuni* genetic complementation plasmid	[1]
pSV010	pSV009 containing *fliW*; donor for *fliW* GC[a] fragment	[1]
pSG001	pSV009 containing *mcp4*_1*;* donor for *mcp4_1* GC fragment	This study
pSG002	pSV009 containing *flaG;* donor for *flaG* GC fragment	This study
pSG003	pSV009 containing *pstA;* donor for *pstA* GC fragment	This study
pSG004	pSV009 containing *fabL;* donor for *fabL* GC fragment	This study
pSG005	pSV009 containing *livM;* donor for *livM* GC fragment	This study
pSG006	pSV009 containing *trxC;* donor for *trxC* GC fragment	This study
pSG007	pSV009 containing *capM;* donor for *capM* GC fragment	This study
pSG008	pSV009 containing *maf3;* donor for *maf3* GC fragment	This study
pSG009	pSV009 containing *flgK;* donor for *flgK* GC fragment	This study

[a] GC, genetic complementation
Abbreviations for antibiotics and plasmids: Cm^r, chloramphenicol resistance (10 µg/ml); Km^r, kanamycin resistance (50 µg/ml).

Table S6 Bacterial strains and plasmids used in this work.

Primer Name	Target [a]	Sequence (5' - 3') [b, c]
PBGSF20	*mariner* Tn element encoding Cm-resistance from pAJG39	ACAGGTTGGATGATAAGTCCCCGGTCT
PBGSF23	Tn-seq amplification	CAAGCAGAAGACGGCATACGAAGACCGGGGACTTATCATCCAACCTGT
PBGSF31	Tn-seq amplification	AATGATACGGCGACCACCGAGATCTACACTCTTTCCCTACACGACGCTCTTCCGATCT
PBGSF29	Tn-seq adapter A	TTCCCTACACGACGCTCTTCCGATCTATCACGNN
PBGSF30	Tn-seq adaptor A	P-CGTGATAGATCGGAAGAGCGTCGTGTAGGGAAAGAGT-P
PBGSF29	Tn-seq adapter B	TTCCCTACACGACGCTCTTCCGATCTCGATGTNN
PBGSF30	Tn-seq adaptor B	P-ACATCGAGATCGGAAGAGCGTCGTGTAGGGAAAGAGT-P
PBGSF29	Tn-seq adapter C	TTCCCTACACGACGCTCTTCCGATCTTTAGGCNN
PBGSF30	Tn-seq adaptor C	P-GCCTAAAGATCGGAAGAGCGTCGTGTAGGGAAAGAGT-P
PBGSF29	Tn-seq adapter D	TTCCCTACACGACGCTCTTCCGATCTTGACCANN
PBGSF30	Tn-seq adaptor D	P-TGGTCAAGATCGGAAGAGCGTCGTGTAGGGAAAGAGT-P
PBGSF29	Tn-seq adapter E	TTCCCTACACGACGCTCTTCCGATCTAGTCAANN
PBGSF30	Tn-seq adaptor E	P-TTGACTAGATCGGAAGAGCGTCGTGTAGGGAAAGAGT-P
PBGSF29	Tn-seq adapter F	TTCCCTACACGACGCTCTTCCGATCTGCCAATNN
PBGSF30	Tn-seq adaptor F	P-ATTGGCAGATCGGAAGAGCGTCGTGTAGGGAAAGAGT-P
PBGSF29	Tn-seq adapter G	TTCCCTACACGACGCTCTTCCGATCTCAGATCNN
PBGSF30	Tn-seq adaptor G	P-GATCTGAGATCGGAAGAGCGTCGTGTAGGGAAAGAGT-P
PBGSF29	Tn-seq adapter H	TTCCCTACACGACGCTCTTCCGATCTACTTGANN
PBGSF30	Tn-seq adaptor H	P-TCAAGTAGATCGGAAGAGCGTCGTGTAGGGAAAGAGT-P
PBGSF29	Tn-seq adapter I	TTCCCTACACGACGCTCTTCCGATCTGATCAGNN
PBGSF30	Tn-seq adaptor I	P-CTGATCAGATCGGAAGAGCGTCGTGTAGGGAAAGAGT-P
PBGSF29	Tn-seq adapter J	TTCCCTACACGACGCTCTTCCGATCTTAGCTTNN
PBGSF30	Tn-seq adaptor J	P-AAGCTAAGATCGGAAGAGCGTCGTGTAGGGAAAGAGT-P
PBGSF29	Tn-seq adapter K	TTCCCTACACGACGCTCTTCCGATCTGGCTACNN
PBGSF30	Tn-seq adaptor K	P-GTAGCCAGATCGGAAGAGCGTCGTGTAGGGAAAGAGT-P
PBGSF29	Tn-seq adapter L	TTCCCTACACGACGCTCTTCCGATCTCTTGTANN
PBGSF30	Tn-seq adaptor L	P-TACAAGAGATCGGAAGAGCGTCGTGTAGGGAAAGAGT-P
PBGSF29	Tn-seq adapter M	TTCCCTACACGACGCTCTTCCGATCTAGTTCCNN
PBGSF30	Tn-seq adaptor M	P-GGAACTAGATCGGAAGAGCGTCGTGTAGGGAAAGAGT-P
PBGSF29	Tn-seq adapter N	TTCCCTACACGACGCTCTTCCGATCTATGTCANN
PBGSF30	Tn-seq adaptor N	P-TGACATAGATCGGAAGAGCGTCGTGTAGGGAAAGAGT-P
PBGSF29	Tn-seq adapter O	TTCCCTACACGACGCTCTTCCGATCTCCGTCCNN
PBGSF30	Tn-seq adaptor O	P-GGACGGAGATCGGAAGAGCGTCGTGTAGGGAAAGAGT-P
PBGSF29	Tn-seq adapter P	TTCCCTACACGACGCTCTTCCGATCTGTCCGCNN
PBGSF30	Tn-seq adaptor P	P-GGACGGAGATCGGAAGAGCGTCGTGTAGGGAAAGAGT-P
PBGSF29	Tn-seq adapter Q	TTCCCTACACGACGCTCTTCCGATCTGTGAAANN
PBGSF30	Tn-seq adaptor Q	P-GCGGACAGATCGGAAGAGCGTCGTGTAGGGAAAGAGT-P
PBGSF29	Tn-seq adapter R	TTCCCTACACGACGCTCTTCCGATCTGTGGCCNN
PBGSF30	Tn-seq adaptor R	P-GGCCACAGATCGGAAGAGCGTCGTGTAGGGAAAGAGT-P
PBGSF29	Tn-seq adapter S	TTCCCTACACGACGCTCTTCCGATCTGTTTCGNN
PBGSF30	Tn-seq adaptor S	P-CGAAACAGATCGGAAGAGCGTCGTGTAGGGAAAGAGT-P
PBGSF29	Tn-seq adapter T	TTCCCTACACGACGCTCTTCCGATCTCGTACGNN
PBGSF30	Tn-seq adaptor T	P-CGTACGAGATCGGAAGAGCGTCGTGTAGGGAAAGAGT-P
L_WITS_1_cam_FW1	Cm^r cassette	AAGCTTGGCGTGGGAGTG

R_WITS_cam_RV1	Cm^r cassette	GAATTCCGCCCTTTAGTTCC
CC069	Cm^r cassette control primer	ATATGTGCAGGGCGTATTGC
0017_L1_FW1	*mcp4*_1 (CJM1cam_0017) left flank	CAAACCAAGAAGATTTAGGGCGC
0017_L2_RV1	*mcp4*_1 (CJM1cam_0017) left flank	CTCACTCCCACGCCAAGCTTTCATCTGAAATTTCTAGTTGTTGAAC
0017_R1_FW1	*mcp4*_1 (CJM1cam_0017) right flank	GGAACTAAAGGGCGGAATTCAATGTTCTAAATGGTGCCTCT
0017_R2_RV1	*mcp4*_1 (CJM1cam_0017) right flank	CCTACAGGCAAGAGTATAATCAA
0017_A_RV1	*mcp4*_1 (CJM1cam_0017) control primer	TGCTTGAACACTTGGATCGT
0049_L1_FW1	*fliK* (CJM1cam_0049) left flank	TGGAACACTTCTTGCTTTAAAG
0049_L2_RV1	*fliK* (CJM1cam_0049) left flank	CTCACTCCCACGCCAAGCTTCTAAATTTGACATCACTTTTCCTTC
0049_R1_FW1	*fliK* (CJM1cam_0049) right flank	GGAACTAAAGGGCGGAATTCAGAAGAAGATACCACAGATACT
0049_R2_RV1	*fliK* (CJM1cam_0049) right flank	TCTCTTTGTTTTGAACTACTTGAG
0049_A_RV1	*fliK* (CJM1cam_0049) control primer	TCTGTTCATCGCTTCACTAACA
0163_L1_FW1	*moaA* (CJM1cam_0163) left flank	TGATAGCGAAGAGGATGAAGACT
0163_L2_RV1	*moaA* (CJM1cam_0163) left flank	CTCACTCCCACGCCAAGCTTAGGCAGCGAAAGTTACATCT
0163_R1_FW1	*moaA* (CJM1cam_0163) right flank	GGAACTAAAGGGCGGAATTCTGGAGTGTTGTTGATAATGAAACC
0163_R2_RV1	*moaA* (CJM1cam_0163) right flank	TTCTATACTTCCATTGCTTTCA
0163_A_RV1	*moaA* (CJM1cam_0163) control primer	TCCTTCGTCTATAGCTGCTT
CJM1_0181_L1	*purN* (CJM1cam_0181) left flank	ACCCCTCATCCAAAAGAATT
CJM1_0181_L2	*purN* (CJM1cam_0181) left flank	CTCACTCCCACGCCAAGCTTTCCATTTCCACTAAAAAGTACAGC
CJM1_0181_R1	*purN* (CJM1cam_0181) right flank	GGAACTAAAGGGCGGAATTCGCCTAGAACATGAAATTTTACCC
CJM1_0181_R2	*purN* (CJM1cam_0181) right flank	ACAATCAAGAAAAGCCCACC
CJM1_0181_A	*purN* (CJM1cam_0181) control primer	TCTTCACGAGTATTATAAGCCT
CJM1_0182_L1	*nnr* (CJM1cam_0182) left flank	AGGAATGGGGCTTGAAAAGTC
CJM1_0182_L2	*nnr* (CJM1cam_0182) left flank	CTCACTCCCACGCCAAGCTTGCTTGGCTAAATTTAATCCTGC
CJM1_0182_R1	*nnr* (CJM1cam_0182) right flank	GGAACTAAAGGGCGGAATTCGCTTTGACGCTTTAAAACTTATC
CJM1_0182_R2	*nnr* (CJM1cam_0182) right flank	CACTCACACCAGCCACTTTC
CJM1_0182_A	*nnr* (CJM1cam_0182) control primer	CAAGCCATCTGAAGCGTTGT
CJM1_0183_L1	CJM1cam_0183 left flank	TGTAGCTATGGATGAGATCAGC
CJM1_0183_L2	CJM1cam_0183 left flank	CTCACTCCCACGCCAAGCTTCCTCTCCGTAATCTGAAGCG
CJM1_0183_R1	CJM1cam_0183 right flank	GGAACTAAAGGGCGGAATTCTTTTCGTGCTTTGATCCAGAAA
CJM1_0183_R2	CJM1cam_0183 right flank	AACGATTTGAACCTGTGCCT
CJM1_0183_A	CJM1cam_0183 control primer	TGCGGATTTGTGCGTATTCA
0239_L1_FW1	*eptA* (CJM1cam_0239) left flank	ACCCTGCCTTCTTCATCATCA
0239_L2_RV1	*eptA* (CJM1cam_0239) left flank	CTCACTCCCACGCCAAGCTTAACTGAAACCAAGTTAATCTAAGCA
0239_R1_FW1	*eptA* (CJM1cam_0239) right flank	GGAACTAAAGGGCGGAATTCGCACTCTTTTAGGATATTTTGATG
0239_R2_RV1	*eptA* (CJM1cam_0239) right flank	AGCCCCAAACAAAAAGAGCT
0239_A_RV1	*eptA* (CJM1cam_0239) control primer	CCACGTGAACAAGGGCAAAA
0297_L1_FW1	*hisC* (CJM1cam_0297) left flank	GCGGCTAAGCTTTATAATGTGC
0297_L2_RV1	*hisC* (CJM1cam_0297) left flank	CTCACTCCCACGCCAAGCTTTCTTTTCCGGGTTCATAATTGC
0297_R1_FW1	*hisC* (CJM1cam_0297) right flank	GGAACTAAAGGGCGGAATTCCGCATTACTATAGGAACATCTTATG
0297_R2_RV1	*hisC* (CJM1cam_0297) right flank	CTTCAAAACCCACGCGACTA
0297_A_RV1	*hisC* (CJM1cam_0297) control primer	TCTATAGCTTTTGGTGGCGT

0303_L1_FW1	CJM1cam_0303 left flank	GCTACTCCGCAAAGACTATGTG
0303_L2_RV1	CJM1cam_0303 left flank	CTCACTCCCACGCCAAGCTTCTAAAAGAGTGCAATCAGATAG
0303_R1_FW1	CJM1cam_0303 right flank	GGAACTAAAGGGCGGAATTCGAAAAATATTGCAAAGATAGGGAT
0303_R2_RV1	CJM1cam_0303 right flank	CCCCTGCTTCTATAAATTCGACA
0303_A_RV1	CJM1cam_0303 control primer	GAGCAAAATTTAAATTCGAAGCA
0368_L1_FW1	*pflB* (CJM1cam_0368) left flank	TCACTAAACCTGAGCAAAGTGA
0368_L2_RV1	*pflB* (CJM1cam_0368) left flank	CTCACTCCCACGCCAAGCTTTCTTGTTCAGCCATCTATACTCT
0368_R1_FW1	*pflB* (CJM1cam_0368) right flank	GGAACTAAAGGGCGGAATTCATGAAGCTGCGATATCTCAG
0368_R2_RV1	*pflB* (CJM1cam_0368) right flank	CATCCTTAGGCTACAGTGGC
0368_A_RV1	*pflB* (CJM1cam_0368) control primer	TCATCAGGCATAACAACAGGT
0438_L1_FW1	*clpB* (CJM1cam_0438) left flank	GCTAGCAGTGCACTTAGAGC
0438_L2_RV1	*clpB* (CJM1cam_0438) left flank	CTCACTCCCACGCCAAGCTTTCCTCTGTAAATAGAAGCTTTTAC
0438_R1_FW1	*clpB* (CJM1cam_0438) right flank	GGAACTAAAGGGCGGAATTCAACGAAACAAATCAACACCTT
0438_R2_RV1	*clpB* (CJM1cam_0438) right flank	TCGTCTTGTTCAACACTTTCA
0438_A_RV1	*clpB* (CJM1cam_0438) control primer	ATCCTTGTTCTTTGGCGTCT
0449_L1_FW1	*glnP* (CJM1cam_0449) left flank	GAGGAAGATGGAGAAATTTTGG
0449_L2_RV1	*glnP* (CJM1cam_0449) left flank	CTCACTCCCACGCCAAGCTTCGTAAGCCAAGCAGCCGTTAA
0449_R1_FW1	*glnP* (CJM1cam_0449) right flank	GGAACTAAAGGGCGGAATTCGGGATATTTGTGTATTATCTTACC
0449_R2_RV1	*glnP* (CJM1cam_0449) right flank	ATACTCTGCAAGGAAACACG
0449_A_RV1	*glnP* (CJM1cam_0449) control primer	AGGCGTATTTCTTGAAAACTCT
0522_L1_FW1	*flaG* (CJM1cam_0522) left flank	GCTTGTTTGGCGTGTGGTAA
0522_L2_RV1	*flaG* (CJM1cam_0522) left flank	CTCACTCCCACGCCAAGCTTTTTGCCCATTTGCCTTCGAT
0522_R1_FW1	*flaG* (CJM1cam_0522) right flank	GGAACTAAAGGGCGGAATTCAGCTGAGTATTTTAGGGATGTGA
0522_R2_RV1	*flaG* (CJM1cam_0522) right flank	ACAAAACCGCTGTCATTTGC
0522_A_RV1	*flaG* (CJM1cam_0522) control primer	CTCGTTGCTGTCCATCATCG
0523_L1_FW1	*fliD* (CJM1cam_0523) left flank	GGAAATATCGAAGGCAAATGGG
0523_L2_RV1	*fliD* (CJM1cam_0523) left flank	CTCACTCCCACGCCAAGCTTAAACCCCAGAACCAAATCCT
0523_R1_FW1	*fliD* (CJM1cam_0523) right flank	GGAACTAAAGGGCGGAATTCCAATGGCGAATCAATGGTTGC
0523_R2_RV1	*fliD* (CJM1cam_0523) right flank	TGCCAAAGATAAAAGTTGAATTTCTC
0523_A_RV1	*fliD* (CJM1cam_0523) control primer	GCTAATGATGAAACAGCCGTCT
0551_L1_FW1	*ilvB* (CJM1cam_0551) left flank	CGATATCAAAGAGGCAATGAAAC
0551_L2_RV1	*ilvB* (CJM1cam_0551) left flank	CTCACTCCCACGCCAAGCTTGCTGAACCGCTTAACTCTTTC
0551_R1_FW1	*ilvB* (CJM1cam_0551) right flank	GGAACTAAAGGGCGGAATTCGATTTTACCAAAGGCAAAGGATAG
0551_R2_RV1	*ilvB* (CJM1cam_0551) right flank	CGATTTGCATCATCAGCAACC
0551_A_RV1	*ilvB* (CJM1cam_0551) control primer	CTGCTTGTTCATGACGCACT
0593_L1_FW1	*pstA* (CJM1cam_0593) left flank	CGAGAAAAGGATTAATGGCGC
0593_L2_RV1	*pstA* (CJM1cam_0593) left flank	CTCACTCCCACGCCAAGCTTGCCCCATTTTACAAAGTCTT
0593_R1_FW1	*pstA* (CJM1cam_0593) right flank	GGAACTAAAGGGCGGAATTCAGTCGTGTTAAATCTTAGTGCG
0593_R2_RV1	*pstA* (CJM1cam_0593) right flank	TTGTCCGCCTGAAAGAGCTA
0593_A_RV1	*pstA* (CJM1cam_0593) control primer	TTTCTTTGTTCTGCGCGAGA
0642_L1_FW1	*rpoN* (CJM1cam_0642) left flank	AAGTGGAGGTGAAAGAAGGC
0642_L2_RV1	*rpoN* (CJM1cam_0642) left flank	CTCACTCCCACGCCAAGCTTTTGCTTGTAAAATCGGCAACC
0642_R1_FW1	*rpoN* (CJM1cam_0642) right flank	GGAACTAAAGGGCGGAATTCAGAATGAAGATCGCAATAAACCT
0642_R2_RV1	*rpoN* (CJM1cam_0642) right flank	CACCAAGTAAACCAAGCGCT

0642_A_RV1	*rpoN* (CJM1cam_0642) control primer	ACTATCCACAAAAGCCGAAT
0784_L1_FW1	*fabL* (CJM1cam_0784) left flank	CTTGCGCACGAACCTAGA
0784_L2_RV1	*fabL* (CJM1cam_0784) left flank	CTCACTCCCACGCCAAGCTTACGAGTTCCACCGCTGATTA
0784_R1_FW1	*fabL* (CJM1cam_0784) right flank	GGAACTAAAGGGCGGAATTCGGTAACAGGACATACTTTCATCGT
0784_R2_RV1	*fabL* (CJM1cam_0784) right flank	GAAGAGCAAGTCCATCTACCC
0784_A_RV1	*fabL* (CJM1cam_0784) control primer	CCAAATCTTGTACCATTTCATCAGC
0894_L1_FW1	*engD* (CJM1cam_0894) left flank	TGCGTTGTAGGACTTGGAGA
0894_L2_RV1	*engD* (CJM1cam_0894) left flank	CTCACTCCCACGCCAAGCTTAGCATTAAAAGTCGTTGATTTGCC
0894_R1_FW1	*engD* (CJM1cam_0894) right flank	GGAACTAAAGGGCGGAATTCGGCGCCAAAGAAGCAGGAAAGC
0894_R2_RV1	*engD* (CJM1cam_0894) right flank	AGGTCATTTGTTGGCTACCGATT
0894_A_RV1	*engD* (CJM1cam_0894) control primer	AGGATAGTTTGCGCTTTGGG
0958_L1_FW1	*jlpA* (CJM1cam_0958) left flank	TCGCCAAATACCCCAATCCT
0958_L2_RV1	*jlpA* (CJM1cam_0958) left flank	CTCACTCCCACGCCAAGCTTCCGCAAGCTGAAAACAAAACA
0958_R1_FW1	*jlpA* (CJM1cam_0958) right flank	GGAACTAAAGGGCGGAATTCAATCAAGACGCCACAGAAGC
0958_R2_RV1	*jlpA* (CJM1cam_0958) right flank	CTTGGGTGGGAGCTTGTTTT
0958_A_RV1	*jlpA* (CJM1cam_0958) control primer	CTGAGAAAGGCTTGCAATTT
0990_L1_FW1	*livM* (CJM1cam_0990) left flank	CAAGTATGGTATAGCTATTCGTGC
0990_L2_RV1	*livM* (CJM1cam_0990) left flank	CTCACTCCCACGCCAAGCTTTTCTAACCATCATCAAAACCTAC
0990_R1_FW1	*livM* (CJM1cam_0990) right flank	GGAACTAAAGGGCGGAATTCTGAGTGATGTGATTAGAGGGATTA
0990_R2_RV1	*livM* (CJM1cam_0990) right flank	TCCAAAACGCCCTAAATGCA
0990_A_RV1	*livM* (CJM1cam_0990) control primer	AGTGAAAATTGTCCAGTTACACCA
1083_L1_FW1	*trxC* (CJM1cam_1083) left flank	AGGAAGTGAAGTTGTAGCCT
1083_L2_RV1	*trxC* (CJM1cam_1083) left flank	CTCACTCCCACGCCAAGCTTTCTTCTTCTTTGGAGCAAGCA
1083_R1_FW1	*trxC* (CJM1cam_1083) right flank	GGAACTAAAGGGCGGAATTCTCCAACAGAACTCATGCAAG
1083_R2_RV1	*trxC* (CJM1cam_1083) right flank	CAAACAGTACATGCTCACAGG
1083_A_RV1	*trxC* (CJM1cam_1083) control primer	CCTTCCTCTCCTTCTACCACA
CJM1_1088_L1	CJM1cam_1088 left flank	ACAGAAGCTCCTTTTAGTGGGA
CJM1_1088_L2	CJM1cam_1088 left flank	CTCACTCCCACGCCAAGCTTGGCAATCCATCACTCATAGCC
CJM1_1088_R1	CJM1cam_1088 right flank	GGAACTAAAGGGCGGAATTCTGCTGCTATGGGGTTTAAGATG
CJM1_1088_R2	CJM1cam_1088 right flank	TCCCTCCCTTAGCTATACGTC
CJM1_1088_A	CJM1cam_1088 control primer	CATCAAGCCCACCATCTTCG
1107_L1_FW1	*capM* (CJM1cam_1107) left flank	TGGAGATGCGGTTGATGAAG
1107_L2_RV1	*capM* (CJM1cam_1107) left flank	CTCACTCCCACGCCAAGCTTCTCAGCGCCTCCTGAATTTA
1107_R1_FW1	*capM* (CJM1cam_1107) right flank	GGAACTAAAGGGCGGAATTCCTAACATCAAAGAAGAATGGCT
1107_R2_RV1	*capM* (CJM1cam_1107) right flank	GCCCTTACCGCTAAATTCCT
1107_A_RV1	*capM* (CJM1cam_1107) control primer	TGTAACTTTAACTTCATTTTCAAGC
1125_L1_FW1	CJM1cam_1125 left flank	AGCCCATGGATTTAAAGGTGA
1125_L2_RV1	CJM1cam_1125 left flank	CTCACTCCCACGCCAAGCTTTGCATATATTCTTTTCTATCTTTGGC
1125_R1_FW1	CJM1cam_1125 right flank	GGAACTAAAGGGCGGAATTCGCCCAAACTTATGTGCAAGA
1125_R2_RV1	CJM1cam_1125 right flank	AGTAAAACAACTTCATAATTACCT
1125_A_RV1	CJM1cam_1125 control primer	CCAACTCTTTTCCATCAACAGC
L1-*luxS*-FW1	*luxS* (CJM1cam_1180) left flank	TTGTGAAATTTGATAGGCTTTAGG
L2-*luxS*-RV1	*luxS* (CJM1cam_1180) left flank	CACTCCCACGCCAAGCTTAGCAGGAGCTGGCATTTTAG
R2-*luxS*-FW1	*luxS* (CJM1cam_1180) right flank	GGAACTAAAGGGCGGAATTCTTGCGCAATGCATTCTTTAG

R1-*luxS*-RV1	*luxS* (CJM1cam_1180) right flank	TCAAGTATAGGTAAGTTCATTTTTGC
C-*luxS*-RV1	*luxS* (CJM1cam_1180) control primer	CGCATTGATAGATATTAAGTTCAGG
CJM1_1213_L1	*kefB* (CJM1cam_1213) left flank	AAGGCAATACGAAAGAGAAGGA
CJM1_1213_L2	*kefB* (CJM1cam_1213) left flank	CTCACTCCCACGCCAAGCTTTCTCCAGCAGCTATATATCCAATGA
CJM1_1213_R1	*kefB* (CJM1cam_1213) right flank	GGAACTAAAGGGCGGAATTCATACGCATCATTCGCTCTGA
CJM1_1213_R2	*kefB* (CJM1cam_1213) right flank	GCTATCGATTAAAGTTCCATCCATAT
CJM1_1213_A	*kefB* (CJM1cam_1213) control primer	GCAAAAGCAAAGGAATAACAGCT
1291_L1_FW1	*maf3* (CJM1cam_1291) left flank	AGTGGGACTAAGTGATCATAGCT
1291_L2_RV1	*maf3* (CJM1cam_1291) left flank	CTCACTCCCACGCCAAGCTTTCAACTTCAAATAAAGCTCGGGT
1291_R1_FW1	*maf3* (CJM1cam_1291) right flank	GGAACTAAAGGGCGGAATTCAAATGGTGGTTACAAAGTTTT
1291_R2_RV1	*maf3* (CJM1cam_1291) right flank	ACAACAATATGCTGATGATTTT
1291_A_RV1	*maf3* (CJM1cam_1291) control primer	AATTCCATTTTCATTTTGCAGT
1412_L1_FW1	*flgK* (CJM1cam_1412) left flank	TGAGAGTGTAACGCCAAGTG
1412_L2_RV1	*flgK* (CJM1cam_1412) left flank	CTCACTCCCACGCCAAGCTTCGATTTGAACTTCGCTTGCC
1412_R1_FW1	*flgK* (CJM1cam_1412) right flank	GGAACTAAAGGGCGGAATTCTCTACCGTAGATCAAATGCTTG
1412_R2_RV1	*flgK* (CJM1cam_1412) right flank	TGGCATCTGTGGTATTTTCTCT
1412_A_RV1	*flgK* (CJM1cam_1412) control primer	AGAATACTCATCATGCAAACGC
CJM1_1436_L1	*zraS* (CJM1cam_1436) left flank	TGATGATTTTGGGTTTTGCTCTT
CJM1_1436_L2	*zraS* (CJM1cam_1436) left flank	CTCACTCCCACGCCAAGCTTATTTCCTTGAACATCAGCTCTTG
CJM1_1436_R1	*zraS* (CJM1cam_1436) right flank	GGAACTAAAGGGCGGAATTCACAAATTATCGAAAGTTTCCAAGGA
CJM1_1436_R2	*zraS* (CJM1cam_1436) right flank	ACCGATATCAATGGAACGCA
CJM1_1436_A	*zraS* (CJM1cam_1436) control primer	CGGTTGCCTCCATTGATGAG
1456_L1_FW1	*fdhA* (CJM1cam_1456) left flank	CGATGGCTTTTATGGAATTTG
1456_L2_RV1	*fdhA* (CJM1cam_1456) left flank	CTCACTCCCACGCCAAGCTTCGGTGTTGCTAGACTTGAAAG
1456_R1_FW1	*fdhA* (CJM1cam_1456) right flank	GGAACTAAAGGGCGGAATTCATGTACGTAAATTTGCAGCG
1456_R2_RV1	*fdhA* (CJM1cam_1456) right flank	TTTTGCCGCTTCTTCACGAA
1456_A_RV1	*fdhA* (CJM1cam_1456) control primer	ATCCCACAGCCTACAGAACAG
1625_L1_FW1	*gltA* (CJM1cam_1625) left flank	AGCTTTCTACTTTTGGACATGGG
1625_L2_RV1	*gltA* (CJM1cam_1625) left flank	CTCACTCCCACGCCAAGCTTCGGAATTTGACATTTTCGCTCC
1625_R1_FW1	*gltA* (CJM1cam_1625) right flank	GGAACTAAAGGGCGGAATTCGGATGGATAGCTCAGTGGATAG
1625_R2_RV1	*gltA* (CJM1cam_1625) right flank	AACGCAAAATTCATCGCTGC
1625_A_RV1	*gltA* (CJM1cam_1625) control primer	TGGGCCAGTTGTACCATCAT
pSV009_GCamplif_FW1	genetic complementation region	TAATAGAAATTTCCCCAAGTCCCA
pSV009_GCamplif_RV1	genetic complementation region	CTATTGCCATAGTAGCTCTTAGTGG
pSV009_seq_FW1	sequencing complemented gene	GGAGACATTCCTTCCGTATCT
pSV009_seq_RV1	sequencing complemented gene	AGCGAGACAAAAACACTGAGC
mcp4.NP.GC.Xho_FW1	*mcp4*_1 genetic complementation	CAGCTCGAGCGGTATTTTTGAAGAATACC
mcp4.GC.Xba_FW1	*mcp4*_1 genetic complementation	CGATCTAGACAGGAGAATATCATGACAAATAAA
mcp4.GC.Bam_RV1	*mcp4*_1 genetic complementation	CGTGGATCCACGTGAAAAAAGTTACTGAAAGCT
*mcp4*_A_RV2	*mcp4*_1 genetic complementation control primer	ACTGCTATGGAATTTGCGGA
fliK.NP.GC.Xho_FW1	*fliK* genetic complementation	CAGCTCGAGCTATAACTGCTATAT
fliK.NP.GC.Xba_RV1	*fliK* genetic complementation	CTGTCTAGATTTAACTTCCTTTTTAAA
fliK.GC.Xba_FW1	*fliK* genetic complementation	CGATCTAGAAATGTCAAATTTAGCTCCGCA
fliK.GC.Bam_RV1	*fliK* genetic complementation	CGTGGATCCCTTTATTTAAACTATCTTGG
*fliK*_A_RV2	*fliK* genetic complementation control primer	GCCCAGATCTTTAGCTATGTCC

flaG.GC.Xho_FW1	*flaG* genetic complementation	GGACTCGAGGATAATTGAGATTTATTTTA
flaG.GC.Bam_RV1	*flaG* genetic complementation	CGTGGATCCACCAAATGCCATTTTTAACTC
fliD.GC.Xho_FW2	*fliD* genetic complementation	GAGCTCGAGTGAGCAAATGGATTCTCTAG
fliD.GC.Bam_RV1	*fliD* genetic complementation	CGAGGATCCTCCTTAATTATTAGAATTGT
*fliD*_A_RV2	*fliD* genetic complementation control primer	ACAAAACCGCTGTCATTTGC
pstA.GC.Xho_FW1	*pstA* genetic complementation	GAGCTCGAGGAAAGCATTCTTATCTCC
pstA.GC.Bam_RV1	*pstA* genetic complementation	CGAGGATCCGCTATCACTTTAATTTTC
fabL.GC.Xho_FW1	*fabL* genetic complementation	CAGCTCGAGGCGCTTTAACTCATTTCGC
fabL.GC.Bam_RV1	*fabL* genetic complementation	GCTGGATCCTTTATTTAAAAGTTGTACCAC
*fabL*_A_RV2	*fabL* genetic complementation control primer	CGCCCACAACAAAAGCATTTAC
engD.NP.GC.Xho_FW1	*engD* genetic complementation	CAGCTCGAGCAAGCGCTATTATGGGAAA
engD.GC.Bam_RV1	*engD* genetic complementation	CGTGGATCCAGGCTATTTTGCCTAAACATTG
*engD*_A_RV2	*engD* genetic complementation control primer	CCTTCGCCTTTACTTGCACC
livM.NP.GC.Xho_FW1	*livM* genetic complementation	CAGCTCGAGGTGTTTCTGGTGTTATC
livM.NP.GC.Xba_RV1	*livM* genetic complementation	CTGTCTAGAAATCTTTCTATTTTTCC
livM.GC.Xba_FW1	*livM* genetic complementation	GCGTCTAGAATGGTTAGAATTAAGGTTTC
livM.GC.Bam_RV1	*livM* genetic complementation	GCTGGATCCGCTTTAACACTACCAAAAC
*livM*_A_RV2	*livM* genetic complementation control primer	ACCAGTCCTCTTGAGCCATT
trxC.NP.GC.Xho_FW1	*trxC* genetic complementation	CAGCTCGAGCACGAAGAAAGAGGTGCTAGA
trxC.GC.Xba_FW1	*trxC* genetic complementation	CGATCTAGAGCTGCTATGAAAAATTATAAATAAAG
trxC.GC.Bam_RV1	*trxC* genetic complementation	CGTGGATCCAGAGTTTGGGTTTTTGGCAT
*trxC*_A_RV2	*trxC* genetic complementation control primer	GCACCATAATCATCCGCGAA
capM.NP.GC.Xho_FW1	*capM* genetic complementation	CAGCTCGAGGGCGATGGGGGGAGTAATTT
capM.GC.Xba_FW1	*capM* genetic complementation	CGATCTAGAGCTTAAAGAGGAGAAATGATGA
capM.GC.Bam_RV1	*capM* genetic complementation	CGTGGATCCAGTTGGCACTATAACAGAAAGT
*capM*_A_RV2	*capM* genetic complementation control primer	ACGCTTCATTACTATGCTCGC
maf3.NP.XhoI_FW1	*maf3* genetic complementation	CAGCTCGAGTAGCACTTGGAGCTAGAATGA
maf3.NP.SpeI_RV1	*maf3* genetic complementation	CTGACTAGTCTAAAATCACTTTCTTTTAAAGC
flgK.GC.Xho_FW1	*flgK* genetic complementation	GAGCTCGAGCAAAGAGTATGCTAAATTTGTTC
flgK.GC.Bam_RV1	*flgK* genetic complementation	CGAGGATCCCATTTGATATTACTAGAGCTTAAG
*flgK*_A_RV2	*flgK* genetic complementation control primer	TGTAAGTCGGGAAAACGCTG
Q-*gyrA*_FW1	*gyrA* (CJM1cam_1001) qPCR primer	TAGGTCGTGCTTTGCCTGAC
Q-*gyrA*-RV1	*gyrA* (CJM1cam_1001) qPCR primer	CGACCTATAACAGCACCCACT
Q-*mcp4*_FW1	*mcp4*_1 qPCR primer	ACGATCCAAGTGTTCAAGCAAT
Q-*mcp4*_RV1	*mcp4*_1 qPCR primer	ACTGCTATGGAATTTGCGGATT
Q-*fliK*_FW1	*fliK* qPCR primer	GGTGCATCAAATCTTAGTGAGCT
Q-*fliK*_RV1	*fliK* qPCR primer	ACATTATCCACAGCACCCTTAAA
Q-*flaG*_FW1	*flaG* qPCR primer	CATTAGCCAAAGAACAAGTGAGACA
Q-*flaG*_RV1	*flaG* qPCR primer	CTCACACCTCGTTGCTGTCC
Q-*fliD*_FW1	*fliD* qPCR primer	GCGATAATCCTCCAGCAAGTC
Q-*fliD*_RV1	*fliD* qPCR primer	AACAAAACCGCTGTCATTTGC
Q-*fliS*_F1	*fliS* qPCR primer	AAAAGGCGGAGAAGTGGCAC
Q-*fliS*_R1	*fliS* qPCR primer	TGCACTTCTCTCCAAGCTTCT
Q-*pstA*_FW1	*pstA* qPCR primer	TGGGGACTCAAAATCTCCTGA
Q-*pstA*_RV1	*pstA* qPCR primer	CCGCTGCAACGCCTATAG
Q-*fabL*_FW1	*fabL* qPCR primer	ATCATTTCAGGACGTGCGGT
Q-*fabL*_RV1	*fabL* qPCR primer	TTTAGCTGCTTCTTGTGCGC

Q-*engD*_FW1	*engD* qPCR primer	TCCTTTCTGCACCATAGAGCC
Q-*engD*_RV1	*engD* qPCR primer	GCACCTTTAACAAGTCCAGCG
Q-*livM*_FW1	*livM* qPCR primer	TGGCTCAAGAGGACTGGTAGA
Q-*livM*_RV1	*livM* qPCR primer	AGCCTTCATTGCACGACCAT
Q-*trxC*_FW1	*trxC* qPCR primer	CACCCCATGCAAAGAAGAAGC
Q-*trxC*_RV1	*trxC* qPCR primer	AGCACCATAATCATCCGCGA
Q-*capM*_FW1	*capM* qPCR primer	CCACTCATCATTAGCGAGCATAG
Q-*capM*_RV1	*capM* qPCR primer	ATACACCTTATCACTGCTTCCAAG
Q-*fliW*-FW1	*fliW* qPCR primer	AGAGCTTCTTTCTTTAACACCTGAATC
Q-*fliW*-RV1	*fliW* qPCR primer	CTGGAGCTAGAAAATTCACTGTCG
Q-*maf3*_FW1	*maf3* qPCR primer	GGAATTTTGTTTATCCTAGCTTCGG
Q-*maf3*_RV1	*maf3* qPCR primer	CAAAGGACGATGCACCAACA
Q-*flgK*_FW1	*flgK* qPCR primer	GCAGCGTTTTCCCGACTTAC
Q-*flgK*_RV1	*flgK* qPCR primer	TTGTGGCATTTTCGTTGGGG

[a] Abbreviations for antibiotics: Cm^r, chloramphenicol resistance. [b] Tn-seq oligonucleotides; barcode sequence for demultiplexing are underlined using a single line. [c] Oligonucleotides for cloning; restriction sites for cloning are underlined using a single line. Primer sequences homologous to Cm^r cassette are underlined using a dotted line for the left flank insert and underlined with a double line for the right flank insert. Cm^r cassette is introduced in between the left and the right flank to construct an insertion-deletion mutant, referred to as defined gene deletion mutants.

Table S7 Oligonucleotides used in this study.

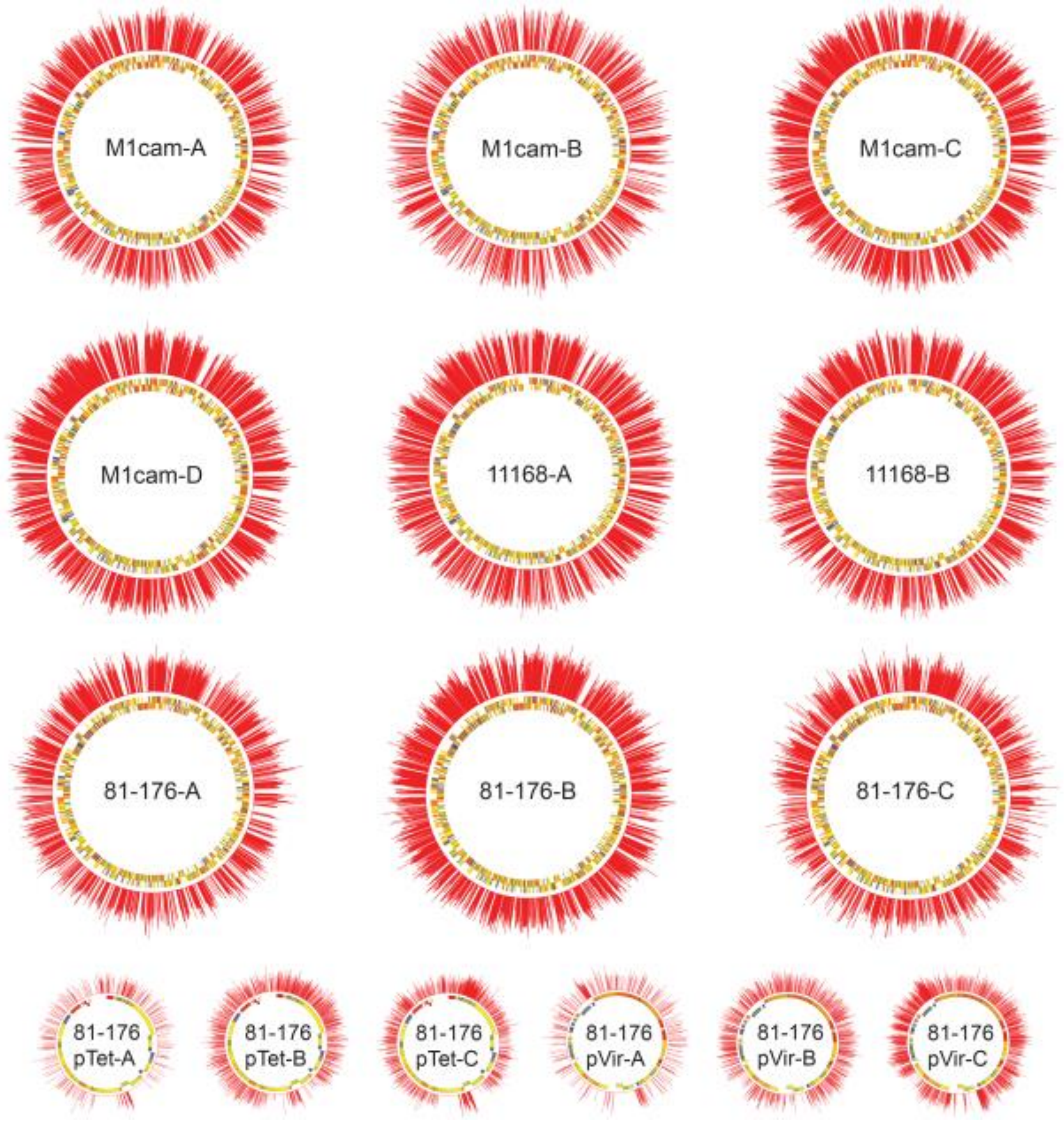

Figure S1. Distribution of Tn insertion sites in *C. jejuni* M1cam, 11168 and 81-176 Tn mutant libraries. Plotted are Log_2 reads per Tn insertion above the read count cut-off (see Table S1 and Methods).

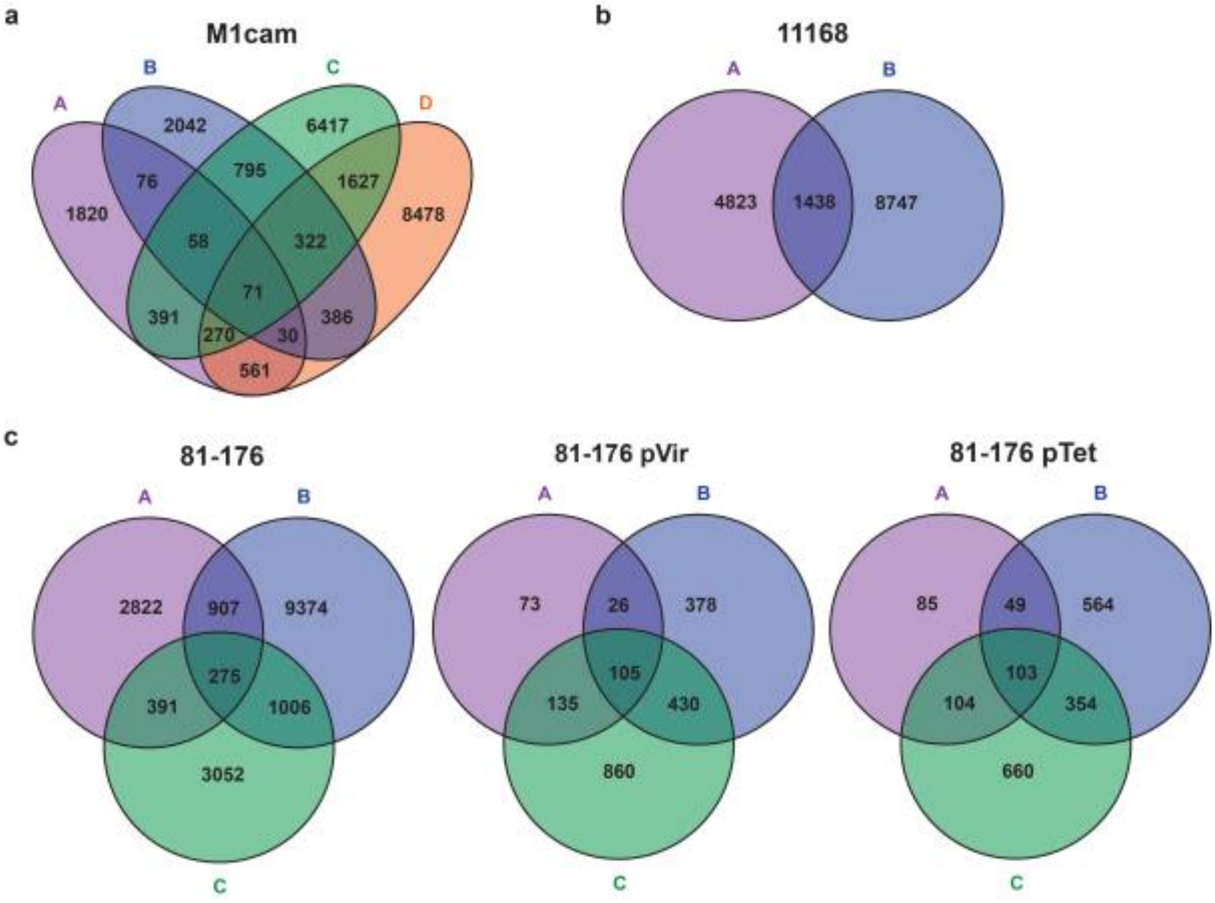

Figure S2. Overlap of the Tn insertions per mutant library in *C. jejuni* M1cam (a), 11168 (b), and 81-176 Tn libraries (c). Only Tn insertion sites above the read count cut-off are included in this analysis (see Table S1 and Methods).

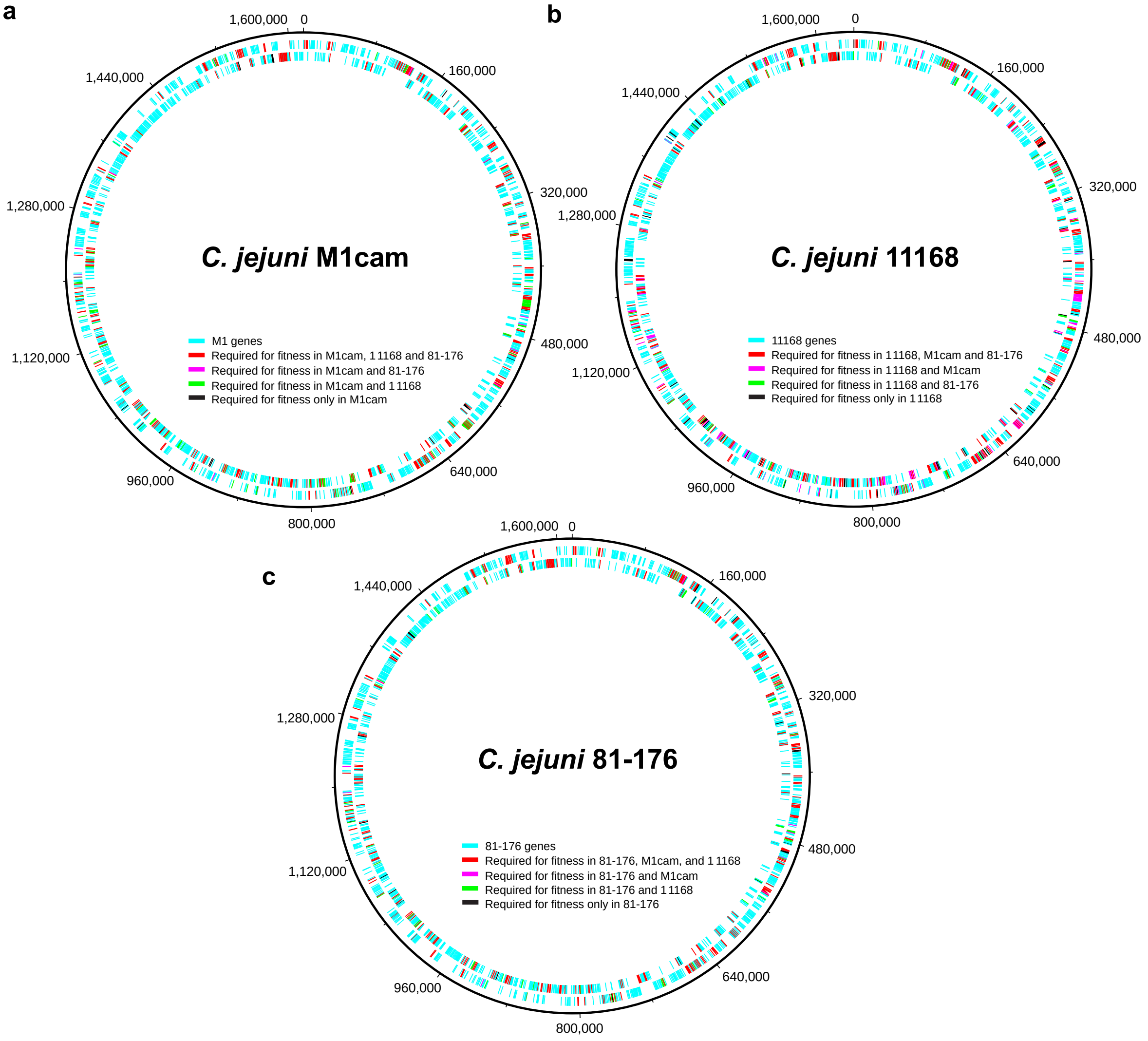

Figure S3. Circular genome visualisation indicating fitness genes in *C. jejuni* M1cam (a), 11168 (b) and 81-176 (c). Genes required for fitness in one, two or all three of the strains are coloured-coded accordingly (See Table S2). A detailed description of the selection criteria is provided in the Methods section.

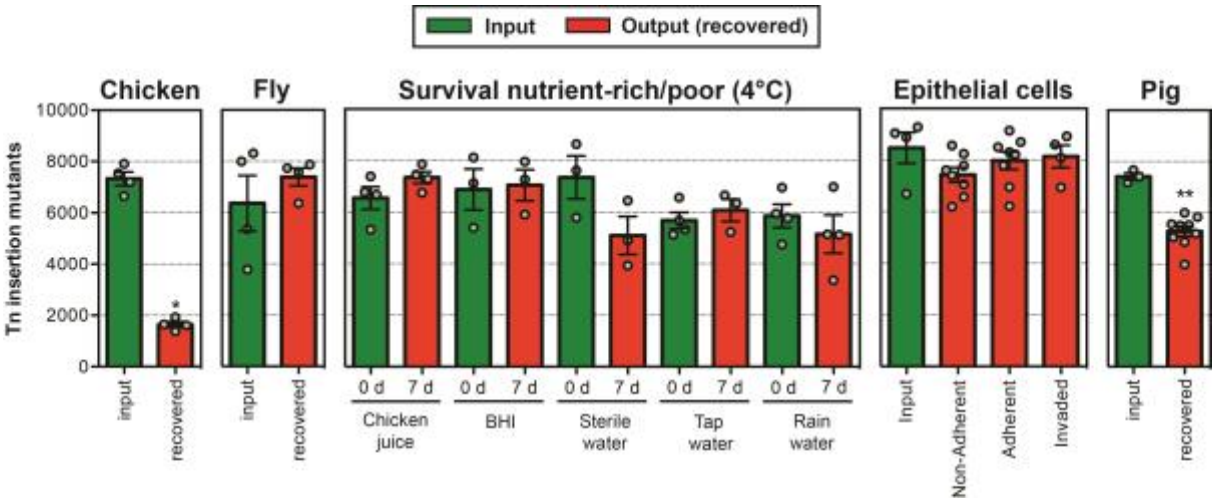

Figure S4. Complexity of the *C. jejuni* M1cam Tn mutant library 'C' (Table S1) in the conditionally essential gene screens as analysed by Tn-seq. Tn mutants represented by > 10 reads were considered to be present. Of note, in the "housefly survival" and "survival under nutrient-rich/poor conditions" screens, a higher number of Tn mutants were detected in the recovered (output) samples, which was the result of a lower sequence depth for some replicates of the input/inocula. Data is shown as individual data points and bars representing the mean with SEM. Statistical significance was analysed using a Mann-Whitney test with * $P < 0.05$ and ** $P < 0.01$.

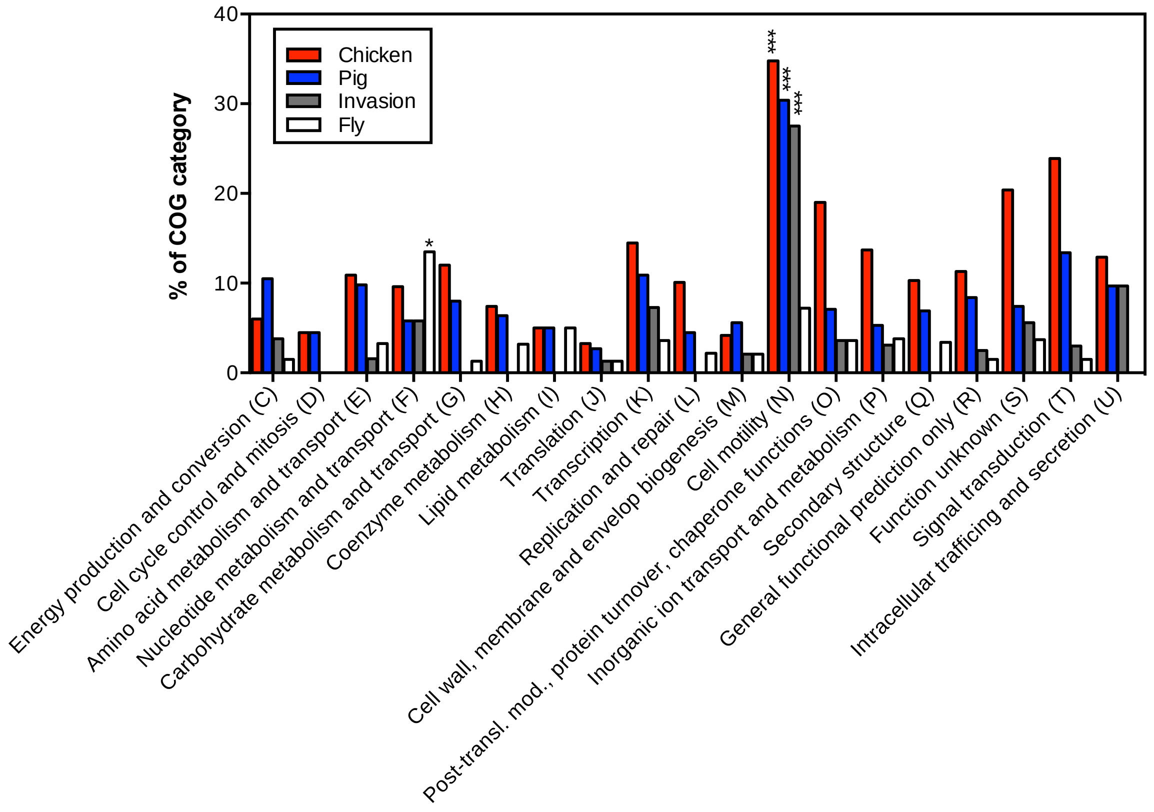

Figure S5. Functional class (COG) enrichment analysis of genes required during colonisation of chickens, infection of gnotobiotic piglets, invasion of human gut epithelial tissue culture cells, or survival in houseflies. The overrepresentation of COG classes were assessed using a Fisher exact test with *Q*-value multiple testing correction; * $Q < 0.05$ and *** $Q < 0.001$.

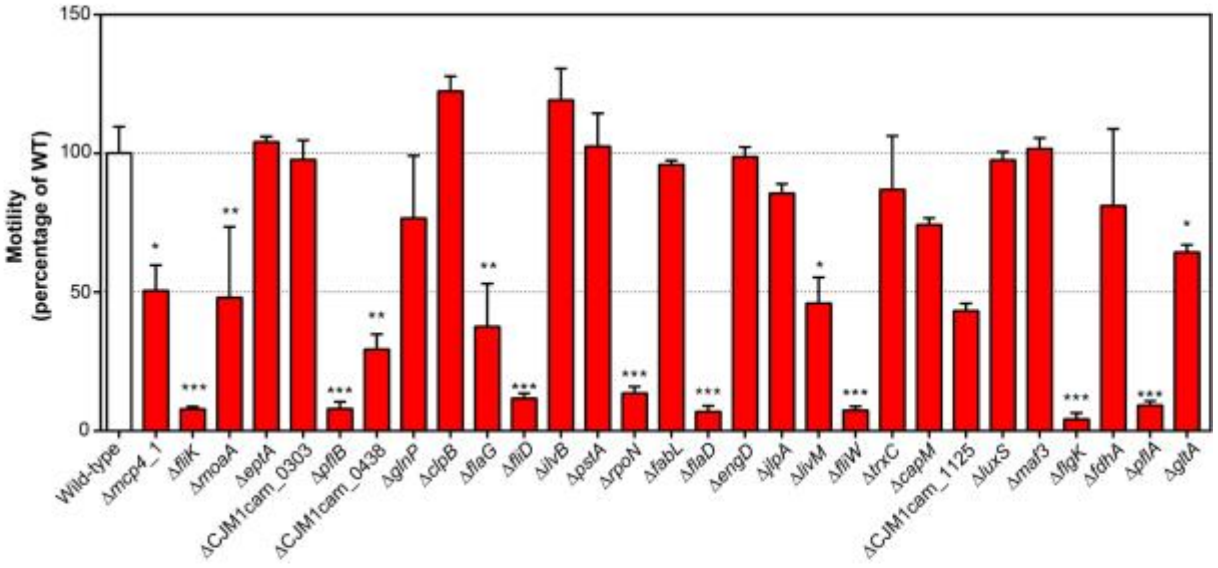

Figure S6. Motility of *C. jejuni* M1cam defined gene deletion mutants. The data is represented as percentage relative to the wild-type ($n \geq 4$). Statistical significance was calculated using Kruskal-Wallis with Dunn's correction for multiple comparisons with * $P < 0.05$, ** $P < 0.01$ and *** $P < 0.001$.

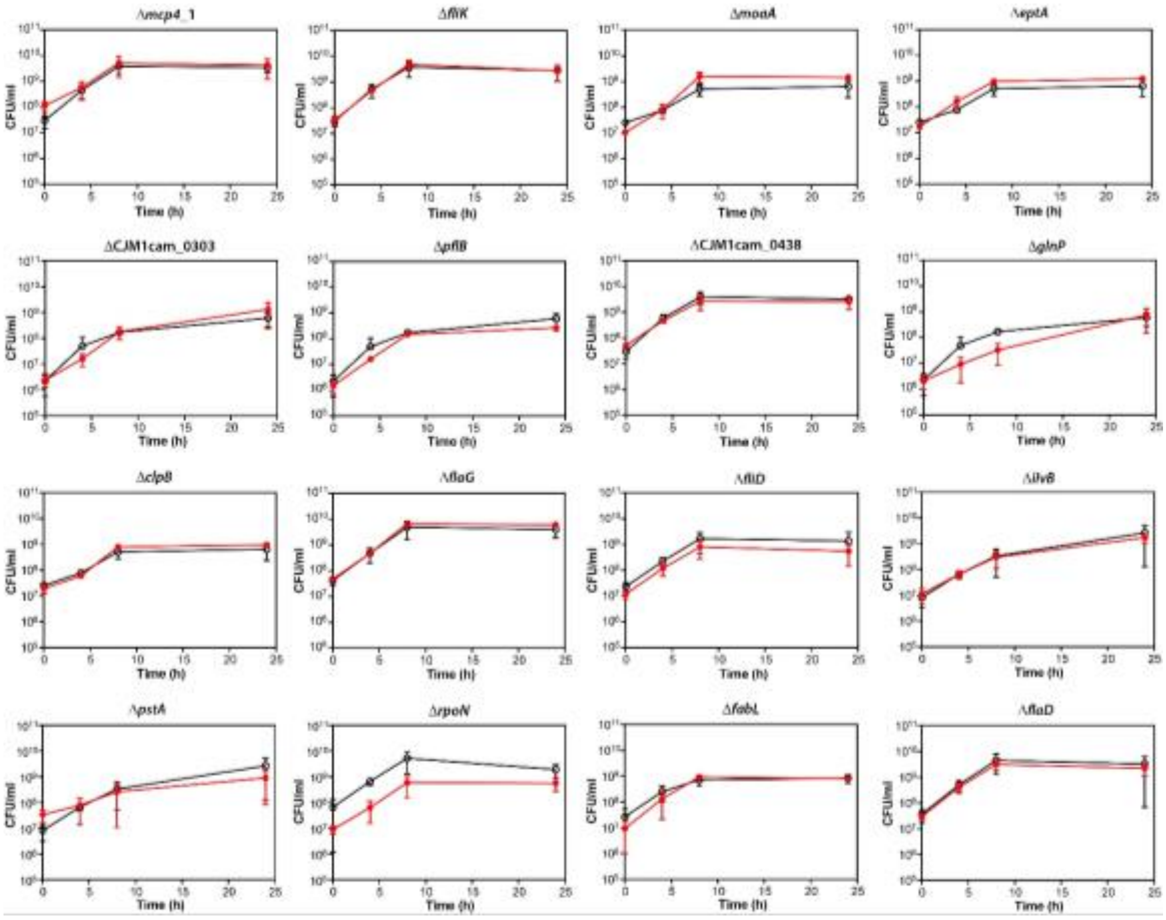
Δmcp4_1
ΔfliK
ΔmoaA
ΔeptA
ΔCJM1cam_0303
ΔpflB
ΔCJM1cam_0438
ΔglnP
ΔclpB
ΔflaG
ΔfliD
ΔilvB
ΔpstA
ΔrpoN
ΔfabL
ΔflaD
CFU/ml
Time (h)

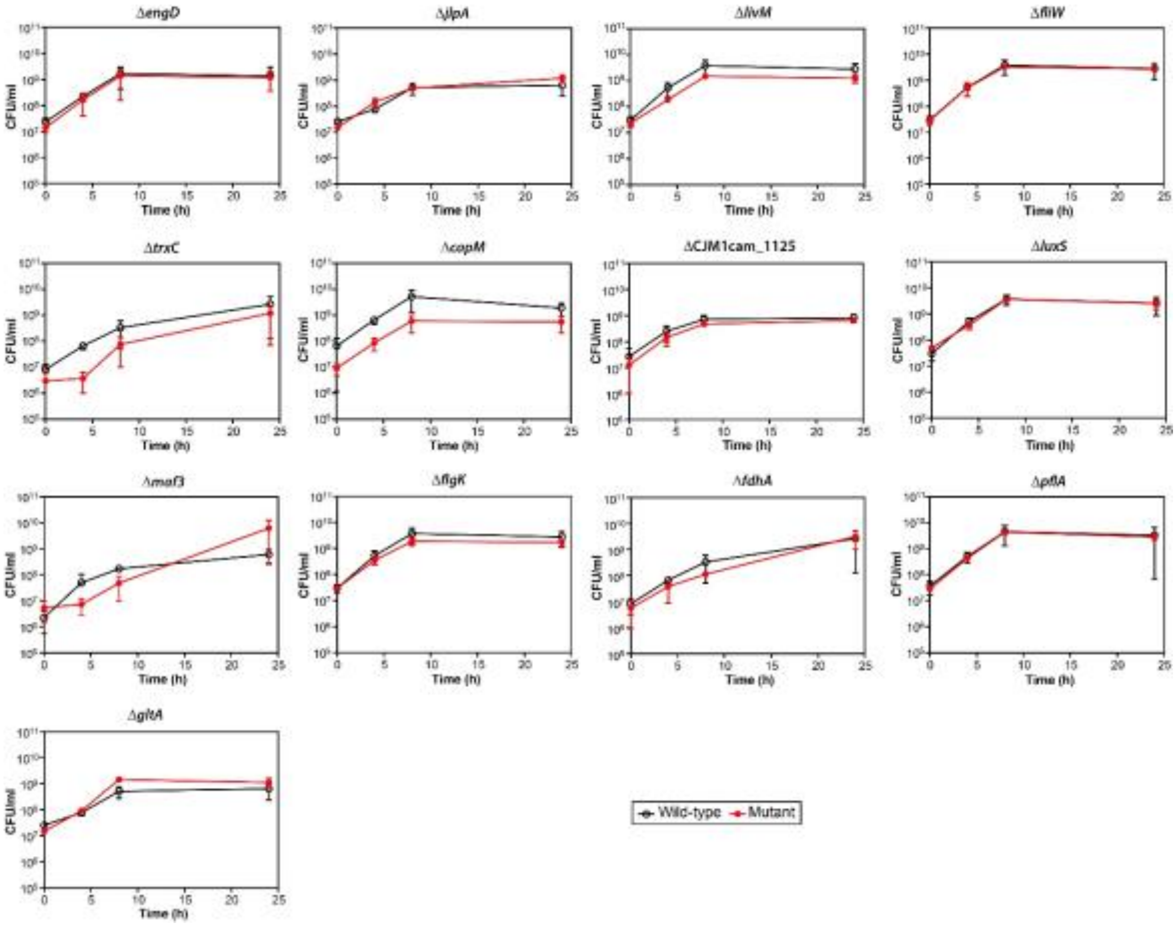
ΔengD
ΔjlpA
ΔlivM
ΔfliW
ΔtrxC
ΔcopM
ΔCJM1cam_1125
ΔluxS
Δmaf3
ΔflgK
ΔfdhA
ΔpflA
ΔgltA
CFU/ml
Time (h)
Wild-type
Mutant

Figure S7. Growth kinetics of *C. jejuni* M1cam defined gene deletion mutants. Growth of *C. jejuni* M1cam wild-type and gene deletion mutants were recorded as viable counts over 24 h at 42°C under microaerophilic conditions. Data is represented as mean and SD ($n \geq 2$).

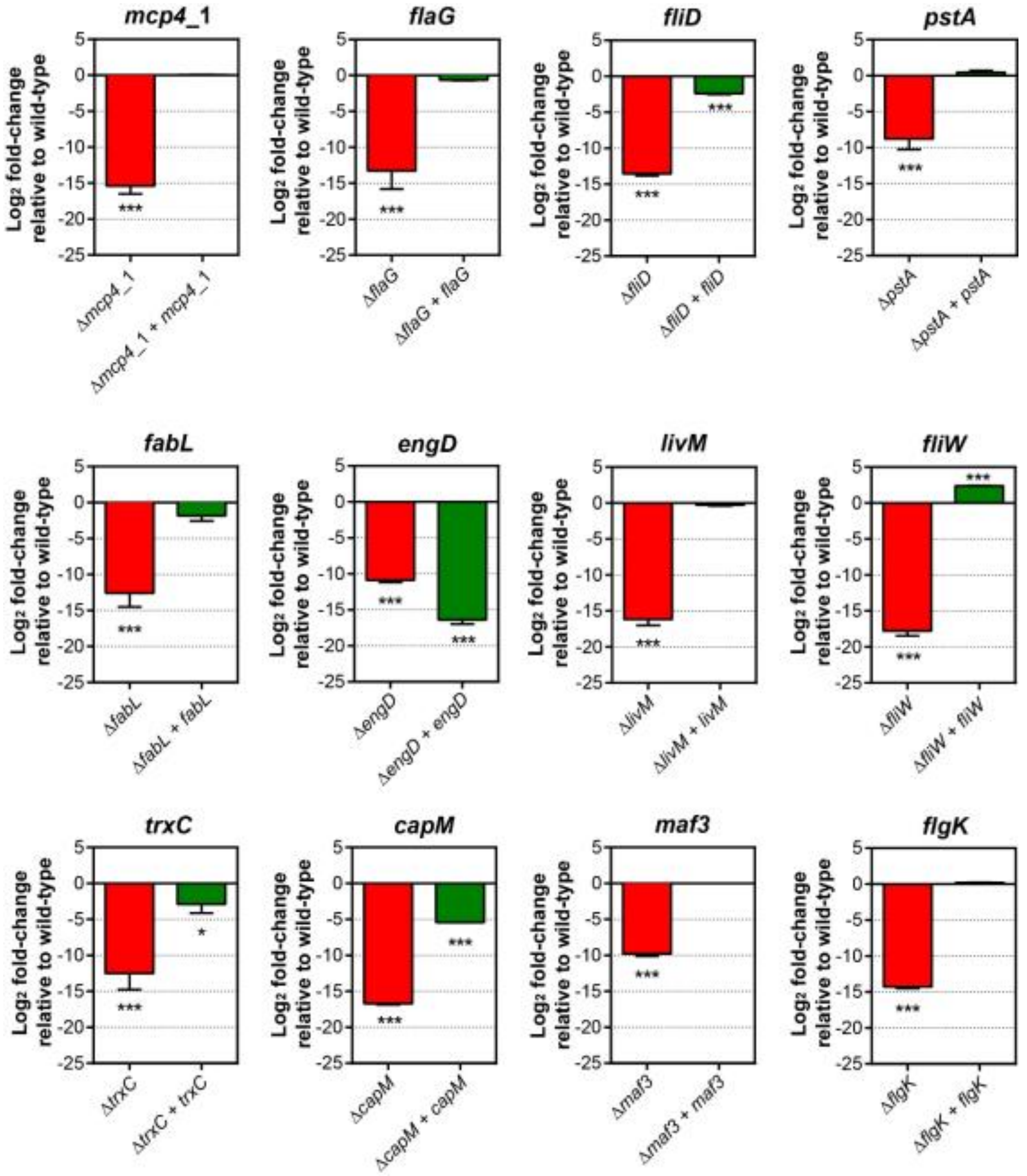

Figure S8. Genetic complementation of *C. jejuni* M1cam defined gene deletion mutants. RT-qPCR analysis revealed the restored expression of *mcp4_1*, *flaG*, *pstA*, *fabL*, *livM*, *fliW*, *maf3* and *flgK* in genetically complemented stains. Lower expression of *fliD*, *trxC* and *capM* was observed in respective genetically complemented strains, compared to the wild-type. Although the *fliD* gene was expressed in the genetically complemented mutant, albeit at lower levels than the wild-type, it was unable to

restore motility (data not shown). This might be (partially) caused by deregulated flagellar assembly due to an increased expression (2.9-fold compared to wild-type) of *fliS* (a flagellar chaperone [8]) located downstream of *fliD* (data not shown). WGS analysis did not reveal any genomic variations in the *fliD* mutant linked to motility (Table S5). The data is represented as Log_2 fold-change relative to the expression levels of the candidate gene in wild-type. Statistical significance ($n \geq 4$) of mutant *vs* wild-type and genetically complemented strains *vs* wild-type was calculated using a one-way ANOVA test with Bonferroni correction for multiple comparisons. For all experiments, data shown are means and SD. * $P < 0.05$, *** $P < 0.001$.

REFERENCES

1 de Vries, S. P. *et al.* Motility defects in *Campylobacter jejuni* defined gene deletion mutants caused by second-site mutations. *Microbiology* **161**, 2316-2327 (2015).

2 Friis, C. *et al.* Genomic characterization of *Campylobacter jejuni* strain M1. *PLoS One* **5**, e12253 (2010).

3 Parkhill, J. *et al.* The genome sequence of the food-borne pathogen *Campylobacter jejuni* reveals hypervariable sequences. *Nature* **403**, 665-668 (2000).

4 Hofreuter, D. *et al.* Unique features of a highly pathogenic *Campylobacter jejuni* strain. *Infect. Immun.* **74**, 4694-4707 (2006).

5 Grant, A. J. *et al.* Signature-tagged transposon mutagenesis studies demonstrate the dynamic nature of cecal colonization of 2-week-old chickens by *Campylobacter jejuni*. *Appl. Environ. Microbiol.* **71**, 8031-8041 (2005).

6 Akerley, B. J. & Lampe, D. J. Analysis of gene function in bacterial pathogens by GAMBIT. *Methods Enzymol.* **358**, 100-108 (2002).

7 Coward, C. *et al.* Competing isogenic *Campylobacter* strains exhibit variable population structures in vivo. *Appl. Environ. Microbiol.* **74**, 3857-3867 (2008).

8 Barrero-Tobon, A. M. & Hendrixson, D. R. Flagellar biosynthesis exerts temporal regulation of secretion of specific *Campylobacter jejuni* colonization and virulence determinants. *Mol. Microbiol.* **93**, 957-974 (2014).
